# Benefits of tropical peatland rewetting for subsidence reduction and forest regrowth: results from a large-scale restoration trial

**DOI:** 10.1038/s41598-024-60462-3

**Published:** 2024-05-10

**Authors:** A. Hooijer, R. Vernimmen, D. Mulyadi, V. Triantomo, Hamdani, M. Lampela, R. Agusti, S. E. Page, J. Doloksaribu, I. Setiawan, B. Suratmanto, S. Swarup

**Affiliations:** 1 Data for Sustainability, 4571 AK Axel, The Netherlands; 2 NUS Environmental Research Institute (NERI), National University of Singapore, 1 Engineering Drive, Singapore, 117411 Singapore; 3 PT Alas Rawa Khatulistiwa, Jl. M.H. Thamrin Kav. 28-30, Jakarta, 10350 Indonesia; 4 Geological Survey of Finland, Vuorimiehentie 5, 02151 Espoo, Finland; 5 Tumbuhan Asli Nusantara, BTN Korpri Blok C1 No. 96, Kawatuna, Mantikulore, Palu, 94233 Indonesia; 6 School of Geography, Geology and the Environment, University of Leicester, Leicester, LE1 7RH UK; 7 Asia Pulp and Paper Group, Sinar Mas Land Plaza, Jakarta, 10350 Indonesia

**Keywords:** Hydrology, Natural variation in plants, Ecosystem services

## Abstract

Drainage and deforestation of tropical peat swamp forests (PSF) in Southeast Asia cause carbon emissions and biodiversity loss of global concern. Restoration efforts to mitigate these impacts usually involve peatland rewetting by blocking canals. However, there have been no studies to date of the optimal rewetting approach that will reduce carbon emission whilst also promoting PSF regeneration. Here we present results of a large-scale restoration trial in Sumatra (Indonesia), monitored for 7.5 years. Water levels in a former plantation were raised over an area of 4800 ha by constructing 257 compacted peat dams in canals. We find peat surface subsidence rates in the rewetted restoration area and adjoining PSF to be halved where water tables were raised from ~ − 0.6 m to ~ − 0.3 m, demonstrating the success of rewetting in reducing carbon emission. A total of 57 native PSF tree species were found to spontaneously grow in the most rewetted conditions and in high densities, indicating that forest regrowth is underway. Based on our findings we propose that an effective PSF restoration strategy should follow stepwise rewetting to achieve substantial carbon emission reduction alongside unassisted regrowth of PSF, thereby enabling the peat, forest and canal vegetation to establish a new nature-based ecosystem balance.

## Introduction

Peat swamps along the coastlines of Southeast Asia cover some 25 million hectares and contain a carbon store that is of global significance^1^. In their natural state these vast wetlands are covered by dense and highly biodiverse tropical rainforest that created the peat from accumulated plant matter over the course of millennia. In recent decades, however, most of these swamps have been deforested and drained, with less than 1 million hectares of intact peat swamp forest (PSF) left in Borneo and Sumatra^2^. Apart from biodiversity loss^3^ this is resulting in major carbon emissions to the atmosphere, partly by fires, that have caused international concern^4^.

In several Southeast Asian countries peatland restoration efforts are being undertaken that involve rewetting and reforestation^5,6^. Rewetting is done by blocking canals to raise water levels. However, there is no clear evidence as to what degree of rewetting will best serve the purpose of forest regrowth, either spontaneous or by seedling planting, and this knowledge gap may have contributed to the limited forest regeneration success in many cases^7^. Moreover, while peatland rewetting is known to reduce carbon emissions based on studies in temperate and boreal climates^8,9^, no published original research evidence exists for this in tropical peatlands.

To help fill this knowledge gap we have set up a large-scale and long-term research project to monitor the response of water table, peat surface subsidence as a measure of carbon emission, and spontaneous tree regrowth to canal blocking. The study was conducted in a former *Acacia crassicarpa* pulp wood plantation on coastal peatland in South Sumatra Province, Indonesia. The area is representative for millions of hectares of domed peatlands across SE Asia that tend to be quite uniform and comparable in many ways, consisting of peat of a woody and fibric nature that has developed in a coastal lowland setting, deforested in recent decades with substantial remnants of natural peat swamp forest remaining nearby.

**Figure 1:**
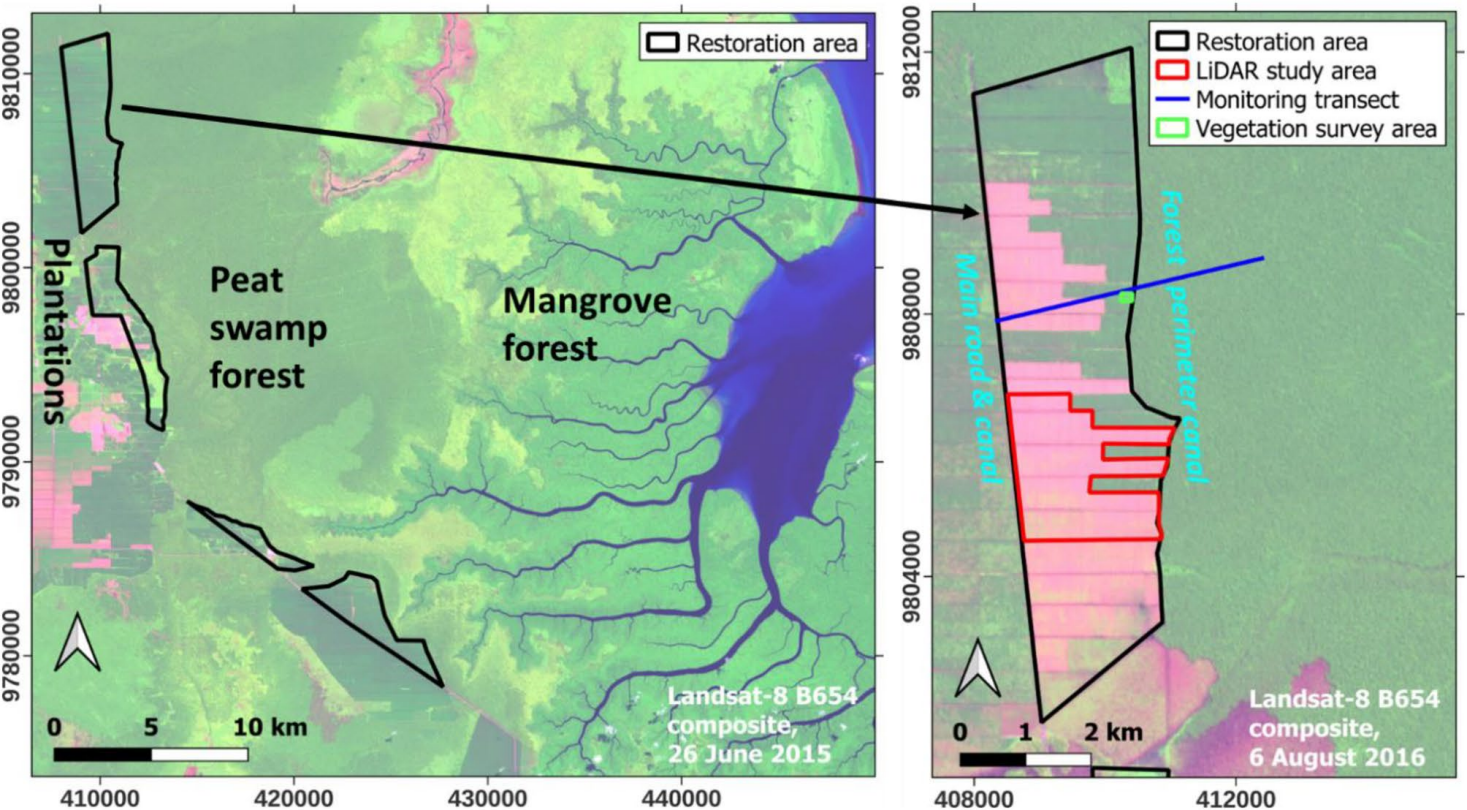
Location of the study area within the broader landscape in South Sumatra, Indonesia. The remaining peat swamp forest (PSF) and mangrove is mostly in Sembilang National Park.

A 35 km long strip of former plantation of up to 2.5 km width along the Sembilang National Park boundary was rewetted to create a protective buffer zone along the remaining intact PSF and mangrove forest. This resulted in a restoration area of ~ 4800 hectares (Fig. 1) with the parallel aims of reducing drainage impacts on the adjoining forest, and creating a research area to help find optimum restoration approaches for carbon emission reduction and unassisted forest restoration.

The decision to retire and rewet this plantation area was taken at quite short notice in preparation for the 2015 El Nino drought which was expected to bring high fire risk. Therefore, only limited pre-intervention data are available for the area and the analysis presented here provides a record of developments during and after the rewetting and restoration process, not a full comparison with initial plantation conditions. Our main research objectives were to [i] determine the actual degree of rewetting that results from canal blocking, [ii] quantify the resulting reduction in peat loss, and [iii] measure forest regrowth in well-rewetted conditions.

## Data and methods

### Study area

The study area has a tropical monsoon climate characterized by high humidity levels, with temperatures ranging from 24.7 to 32.9 °C and average annual precipitation is 2623 mm^10^. The results reported here apply to a central area within the northern part of the wider Tripupa Jaya (TPJ) restoration area i.e. former plantation (Fig. 1), where relatively stable water management conditions were maintained throughout the study period, least affected by management changes in the adjoining plantation on the other side of the main road bordering the area on the West side, where water levels are sometimes adjusted for flood prevention and around harvest cycles. Peat thickness in the area is 7–10 m, with sandy clay underneath.

The restoration intervention consisted of rewetting by canal blocking with compacted peat dams and clearing part of the *A. crassicarpa* plantation cover, that was planted in 2012–2013 when the plantation was established (drainage and clearing of the original forest occurred in 2011–2012). No trees were planted outside of specific research plots, for which results will be reported separately. The N–S perimeter canal between the restoration area and adjacent PSF was blocked in 2015, with blocking of E–W canals inside the restoration area following in 2017 to further raise water levels and rewet the peat. The *A. crassicarpa* plantation crop was harvested in parts of the area from March–May 2016, creating a mosaic of cleared areas amid well-defined areas of remaining *A. crassicarpa* plantation cover (Fig. 1). Both cleared and uncleared areas were included in the current analysis of tree regrowth. Developments in water table depth and forest regrowth in the area were monitored in the field and by airborne LiDAR.

### Canal blocking method for rewetting

A total of 257 dams blocking canals within and surrounding the 4800 ha restoration area were constructed from compacted peat (Supplementary Fig. 5). Each dam measures 8 m across canals and 6 m width, with the crest being above the surrounding peat surface by at least 0.5 m to prevent overflowing and erosion. The peat for dam construction was collected from around the dam site and compacted by excavators, the test of full compaction being an excavator crossing the dam without causing further compaction, at which point the density of the peat was about 2–3 times the original value.

Dams were created at 500 m intervals along all canals. In locations along the Western border of the study area along a main road, bypasses (‘spillways’) were constructed around the dam; these are smaller shallow canals that aim to prevent flooding of the road. Bypass bottom depth was designed to keep canal water levels from dropping more than 0.5 m below the surrounding peat surface.

### Canal monitoring

In selected canals, water levels between just upstream and downstream of blocks were measured at a monthly basis during 2021–2023. Moreover, the canal condition near dams was monitored in all canals at the start and end of the period. This included vegetation species occurrence on the dam, in the canal and along the canal sides. Orthophotos to determine open water area and vegetation type along all canals were collected by drone. In the canal, water flow direction and velocity were estimated using a float device.

### Groundwater table depth and subsidence monitoring in the field

Monitoring was focused on a 4.2 km transect (Fig. 1), that was designed to be representative for the study area by being placed on peat of average depth and nature and by crossing all types of landscape elements in the wider study area including forest, restoration area with different harvesting histories, and different canal drainage regimes. Along this transect, monthly measurements of groundwater table depth (GWD) and peat surface subsidence were conducted from January 2016 in dipwells placed by mid-2015, made of PVC that were anchored in the mineral subsoil for stability. Dipwells were mostly placed at 250 m intervals along the transect, with higher density around the perimeter canal that separates the restored former plantation from the forest. Pronounced hummocks and hollows were avoided during installation, the aim being to place dipwells on surfaces that approach the average peat surface elevation. A few data gaps occurred over time due to periodic accessibility limitations related to COVID-19 and the presence of tigers in the area. Short gaps in isolated dipwell records were filled with data from neighbouring locations with similar water table depth if available, or by interpolation of data before and after the gap at the same location. Outlier values caused by likely transcription errors were corrected or removed. This outlier correction/removal and gap filling amounted to less than 10% of the records overall. Some dipwells were damaged over time. After screening for data availability and quality, data from 20 dipwells is used in this analysis, along 4.2 km of transect, 2.1 km across both the restoration area and forest. This data was further divided into 6 zones of 700 m each, for which mean water table depth and subsidence rate were determined.

### Canal water table depth measurement by LiDAR as an indicator of groundwater table depth

Canal water table depth (CWD) below the nearby peat surface was derived from airborne LiDAR data as described by Vernimmen et al.^11^ for the same area. For the landscape studied here, such data was available at four dates: 25 January 2017, 16 October 2017, 21 August 2018 and 18 July 2020. The first two dates are in the wet season when the canal water table tends to be high, the latter in the dry season when it generally is low. The data therefore provides some coverage of water table depth variation in time, while capturing spatial variation as well. Based on field surveys (Supplementary Note 2) we find that GWD is close to the CWD values. We estimate GWD from CWD as: *GWD* = *CWD* + 0.05 m. This limited difference is explained by the closely spaced field drains (< 200 m intervals) and the high hydraulic conductivity of the peat (119 m d^−1^; Supplementary Note 3) that ensures that excess rainfall is discharged from the peat to canals within days, rapidly equalizing water table levels in the peat and in canals.

### Vegetation monitoring by remote sensing: vegetation height

Vegetation height maps for the study area were created using airborne LiDAR data collected in January 2017, October 2017, August 2018 and July 2020; specifications of LiDAR data collection can be found in Vernimmen et al.^12^. The LiDAR data processing was carried out using PDAL 2.5.6^13^ and PointCloudRasterizers 0.2.5^14^. After removal of data outside the 5 and 65 m + MSL range or having a scan angle greater than 20 degrees, noise was removed using the statistical outlier method^15^. The progressive morphological filter algorithm^16^ was used to classify ground returns and the height above ground nearest neighbour filter was used to determine median vegetation height at 100 m resolution.

For areas where *A. crassicarpa* plantation cover was cleared by 2016, the vegetation height change pattern by 2018 and 2020 was determined to quantify vegetation growth rates from bare soil at 1.5 and 3.5 years after clearing. These growth rates were analysed in relation to the mean of GWD values over the same periods, for an area of 388 ha where GWD could be best estimated from CWD.

### Forest regrowth observations on the ground

Thirty-four 50 m^2^ circular plots (3.99 m radius; total survey area 0.17 ha) near the transect in the ‘unharvested’ part of the restoration area (Fig. 1, Supplementary Fig. 6) and within 150 m from nearby forest were established and surveyed in April 2021 to provide an example of species richness and size of native tree regrowth in optimum conditions, 9 years after the area was cleared of original forest and planted with *A. crassicarpa*. All tree stems with a diameter at breast height (DBH) of more than 1 cm (i.e. excluding seedlings and saplings) were measured with a diameter tape and tree species determined, including *A. crassicarpa*. For randomly selected trees, height of trees smaller than 10 m was measured using a levelling rod, taller trees were measured using a clinometer. The height of all measured trees was then estimated based on the relationship with DBH.

## Results

### Canal blocking

**Figure 2:**
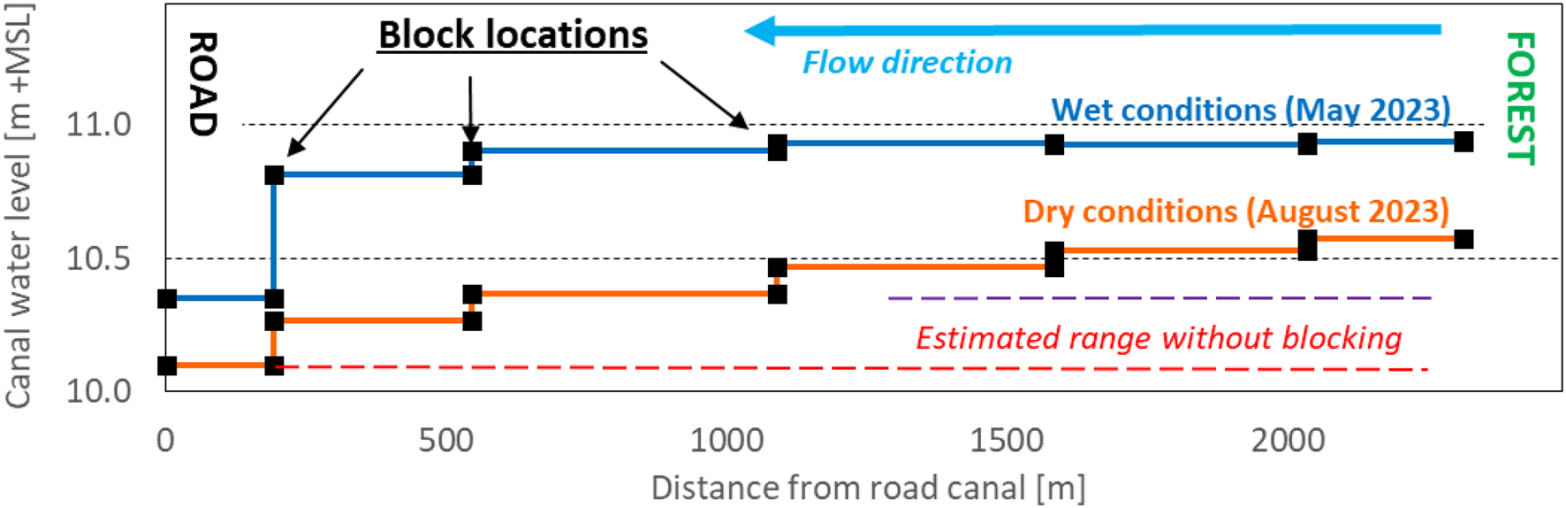
Profile along a blocked E-W canal across the restoration area, near the dipwell transect, showing measured water levels in different months with high (‘wet’) and low (‘dry’) water levels. Water levels were raised the most towards the East of the restoration area, as water flow direction was towards the West. Detailed values for individual canal blocks are presented in Supplementary Note 1 (Supplementary Table 1).

Dams made of compacted peat were observed to be quickly overgrown with vegetation, adding to their stability; indeed all dams (built by 2015–2017) were still intact and functioning 6–8 years later by 2023, without maintenance being required. Water levels were estimated to be raised by some 0.5 m on the East side of the rewetting area (Fig. 2). However, the bypasses that had been created around some dams near the main road canal to the West were found to erode, increasingly reducing the blocking effect which resulted in the gradual loss of rewetting impact. It was therefore decided to close most bypasses in 2023, except those directly along the access road where flood prevention remained a priority.

### Canal vegetation

In canals that were fully blocked by peat dams without bypasses, rapid aquatic vegetation growth was observed, usually starting with floating vegetation followed by grasses and sedges (Supplementary Fig. 7). Woody vegetation was still scarce 5 years after blocking, and limited mostly to canal sides, but increasing. Overall, open water area in fully closed canals (without bypasses) had decreased to 26% on average by 2022–2023 with a range of 7–45%. However, canals with bypasses were far less covered by vegetation as variations in flow velocities were greater.

### Groundwater table depth variations in space and over time

**Figure 3:**
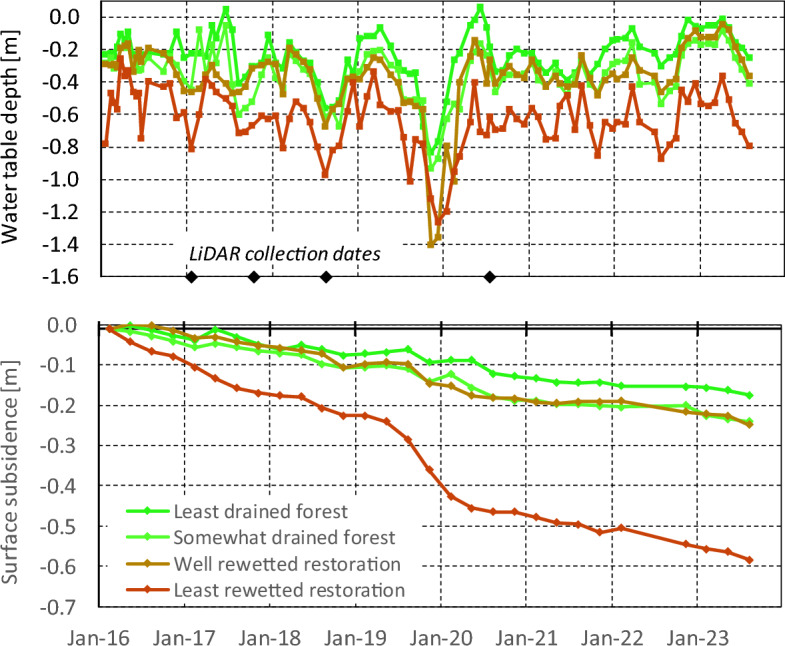
Time series of groundwater table depth (GWD) and peat surface subsidence across the restoration area. Area descriptions in Table 1. LiDAR collection dates refer to Fig. 5.

**Figure 4:**
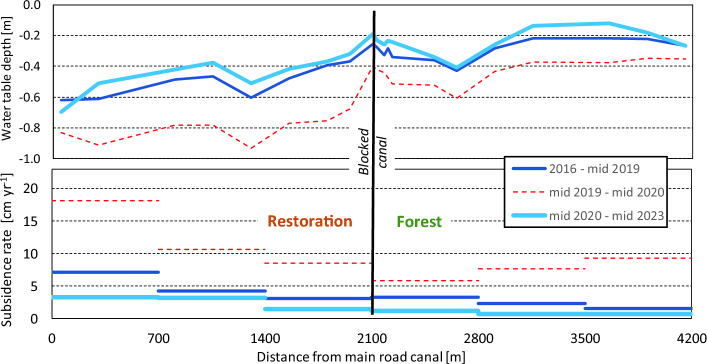
Profiles of groundwater table depth (top) and peat surface subsidence rate (bottom) across the restoration area and 2 km into the forest. Profiles for groundwater table depth and subsidence rate are shown for different periods over the 7.5-year record, to demonstrate the effects of rewetting and drought events.

A clear and consistent gradient in GWD along the transect is observed with the lowest water tables i.e. greatest GWD of − 0.63 m on average over 7.5 years occurring over a ~ 700 m zone near the main road canal and the highest water tables of − 0.25 m occurring in the least drained forest (Table 1, Figs. 3, 4). In the best-rewetted former plantation and most drained forest, intermediate mean GWD of − 0.35 m was measured. In all zones, the ‘normal’ range of GWD between 10 and 90 percentiles, i.e. excluding the driest and wettest 10% of the time, was consistently around 0.4 m, indicating that water table fluctuations are quite uniform even if mean GWD values are highly variable. However, extreme GWD values were well outside of that range, from well below 1 m during the 2019 El Nino drought in the rewetted restoration area to around the peat surface following heavy rainfall in the wet season. Even in the least drained forest zone, GWD dropped below − 0.8 m during the El Nino drought.

**Table 1:**
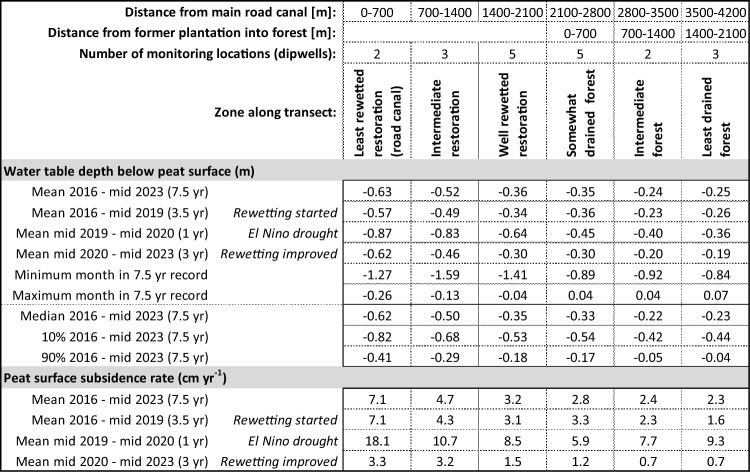
Peat groundwater table depths (GWD) and subsidence rates over zones along the transect through the restoration area, from the main road canal where drainage remains most severe to forest that is ~ 2 km from any drainage.

Values are determined over different periods, and averaged over multiple observation dipwells in each zone. Detailed values for individual dipwells are presented in Supplementary Note 1 (Supplementary Table 2).

A rise in mean water table, i.e. reduction in GWD, is evident in most rewetted locations when comparing values over the first 3.5 and last 3 years on record (Table 1); changes by 2–4 cm or some 10% of mean GWD are seen in the restoration area furthest from the main canal as well as throughout the forest. This limited change must be interpreted in view of the greatest rewetting intervention, i.e. the closure of the perimeter canal between plantation and forest, being constructed in 2015 i.e. before measurements started. The exception to the trend of rising water levels is the zone closest to the main road canal where dams were constructed with bypasses that gradually eroded, before being fully closed early 2023.

In the forest 1400–2100 m away from the perimeter canal, which we consider to be near-natural conditions, the 7.5-year mean GWD value in PSF measured in three dipwells more than 1400 m from the former plantation and perimeter canal, i.e. least likely to be affected by drainage, is ~ 0.25 m with a value of − 0.19 m over the last 3 years on record when rewetting in the restoration area was complete (Table 1). The median 7.5 year value is − 0.23 m with a ‘normal’ range as defined by 10th and 90th percentile values of − 0.44 to − 0.04 m, meaning higher and lower values occur for about one month a year on average each, with extremes reaching − 0.84 and + 0.07 m. Water tables above the peat surface were observed for 4% of the time, corresponding with an average duration of surface inundation of 2 weeks per year, although unevenly distributed with no inundation occurring in several years.

### Peat surface subsidence variations in space and over time

Surface subsidence rates were highly variable along the transect, with mean values over the 7.5-year monitoring period varying from 7.1 cm yr^−1^ near the main road canal where GWD remained greatest to 3.2 cm yr^−1^ in the most rewetted former plantation and 2.2 cm yr^−1^ in the part of the forest where GWD was least, around 2 km away from canals (Table 1). Overall, the relation between subsidence rate and GWD was similar to that found in other longer-term datasets in nearby peatlands (Supplementary Note 4, Supplementary Fig. 1).

The variation over time was equally great, with an overall trend seeing subsidence being reduced by more than half as the system adjusted after the start of rewetting in 2015, with additional rewetting interventions in 2017 and early 2023. Over the first 3.5 years (2016–mid 2019) and last 3 years (mid 2020–mid 2023) on record, subsidence rates in the most-rewetted part of the former plantation dropped from 3.1 to 1.5 cm yr^−1^ which is nearly equal to nearby somewhat drained forest which saw a drop from 2.7 to 1.2 cm yr^−1^ over that same period. Near the main road canal, however, where rewetting was less successful, subsidence rates dropped to 3.1 cm yr^−1^ (from 7.1 cm yr^−1^) which is not much lower than what is reported for fully drained plantations on tropical peatlands elsewhere^17,18^. In forest 2 km from the former plantation the decline in subsidence rate was from 1.4 to 0.7 cm yr^−1^.

In addition to this overall trend of declining subsidence rate, however, we also saw a sharp acceleration of subsidence rate during and shortly after the 2019 El Niño drought event when GWD values were very low, with the rate over mid-2019 to mid-2020 period being 2.5–4 times higher than in the preceding 3.5 years in all land cover types and GWD conditions, and more than double the 7.5-year mean (Fig. 4, Table 1).

### Vegetation height and groundwater table depth monitoring by LiDAR

**Figure 5:**
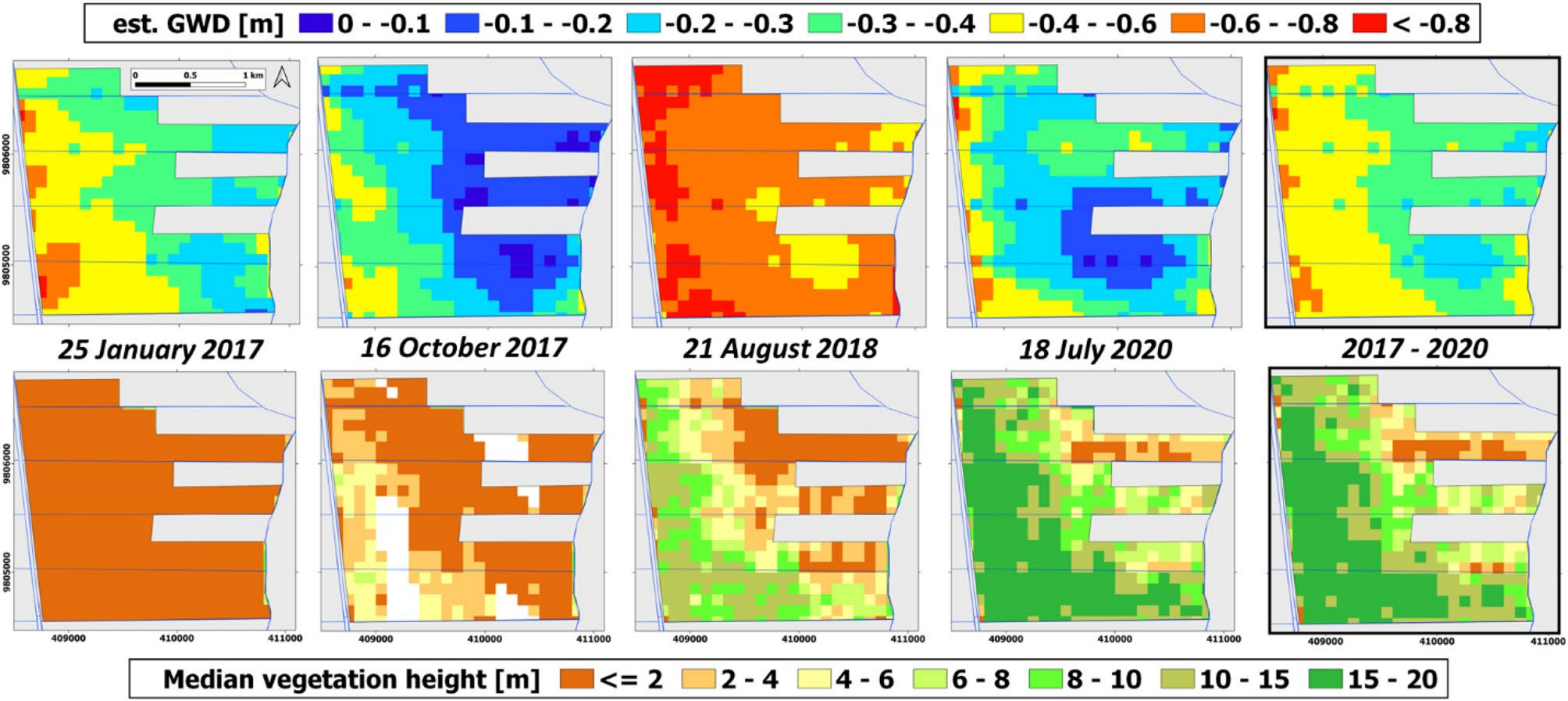
(top panel) estimated groundwater depth (GWD) and (lower panel) median vegetation height as determined from LiDAR collected at 4 dates. The 2017–2020 maps on the right side presents the mean estimated groundwater depth and the total vegetation height growth over a 3.5-year period.

Considerable variation in vegetation height is measured as trees grow (Fig. 5), and a relation with GWD is found especially in the early years of development (Supplementary Note 5, Supplementary Fig. 2). In most of the area, vegetation height exceeds 15 m by July 2020, 3.5 years after the first measurement in January 2017. Field observations and orthophoto inspection confirm that the top canopy consists entirely of *A. crassicarpa* that, while removed from most of the area, spontaneously re-establishes and outgrows all other tree species. However, in almost half the area regrowth is below 10 m and in parts it is below 4 m, suggesting that *A. crassicarpa* as well as other species have difficulty developing in some locations.

The estimated GWD map (Fig. 5) for the LiDAR analysis area also shows considerable spatial variation with mean grid values over 4 measurements in different seasons (January 2017–July 2020) ranging from − 0.24 to − 0.67 m. The overall mean GWD value over all cells and measurements is − 0.41 m, with a temporal range of − 0.08 to − 0.90 m (Fig. 5). In general, a water table depth gradient is observed from the West where lowest GWD values are observed near the road canal to the East, closest to the forest where canal blocking has yielded the greatest rewetting impact.

### Native tree species and size in rewetted peatland

The vegetation regrowth monitoring area was located along the section of the main research transect where rewetting yielded the highest water levels, but without resulting in prolonged inundation (Figs. 1, 4), yielding results that are representative of restoration on rewetted peat.

In the former plantation that remained unharvested for 9 years, *A. crassicarpa* was still a common tree species occurring in 50% of the plots (Supplementary Table 5). However, a total of 57 native tree species were encountered of which 12 were in at least 9 of the 34 plots (at least 25%; Table 2). These 25% of species account for 75% of the overall native tree count of 3600 (Table 2), indicating these other species are dominant overall. On average, plots had 9 native species with a range of 1 to 15.

**Table 2:** Key parameters for the most common native tree regrowth species, occurring in at least 25% of rewetted survey plots (~ 9 years after *A. crassicarpa* planting, ~ 5 years after rewetting).

Species name (most common native species)	Occurring in # plots	Total number of trees*	Mean tree density^$^	Mean stem diameter	Max. stem diameter	Mean tree height	Max. tree height
	[%]	[#]	[# ha^−1^]	[cm]		[m]	
*Ridsdalea grandis*	65	53	312	2.8	6.0	4.7	7.0
*Timonius flavescens*	59	46	271	2.5	5.4	5.3	8.5
*Syzygium incarnatum*	53	65	382	3.0	6.8	5.2	10.0
*Ilex hypoglauca*	50	27	159	2.6	5.1	3.7	6.0
*Planchonella maingayi*	47	54	318	3.7	13.6	6.2	12.0
*Gluta aptera*	47	42	247	3.2	8.2	4.9	9.0
*Elaeocarpus mastersii*	44	54	318	7.9	18.2	10.8	19.8
*Alstonia angustifolia*	38	50	294	7.1	16.0	9.1	15.0
*Ilex cymosa*	35	19	112	2.7	5.8	4.0	6.5
*Tristaniopsis merguensis*	29	17	100	2.7	4.5	6.0	9.0
*Elaeocarpus griffithii*	29	12	71	4.6	8.7	5.3	9.5
*Madhuca motleyana*	26	21	124	2.1	4.7	4.0	7.0
TOTAL COMMON SPECIES (# = 12)	> 25	460	2706	3.7	8.6	5.8	9.9
TOTAL ALL NATIVE SPECIES (# = 57)	> 2	643	3600	3.8	6.1	5.6	7.4

Ranked by frequency of occurrence in plots. The total of all native species counts is added for comparison, see detailed results in Supplementary Table 5. *Most trees have multiple stems, see Supplementary Table 5. ^$^Mean tree density is calculated over all plots, i.e. including plots where the species did not occur. Stem diameter measured from breast height (DBH), mean tree height from height measurements from sample trees of the species.

Of the native tree species encountered in the monitoring area, 87.7% were observed to occur in the PSF that is a few hundred metres away, and we may assume this applies to the other species as well. Seed dispersion is by wind for 9 of the native species encountered, but 52 are dispersed by animals (birds and mammals, including bats) (Supplementary Table 5).

Observed native tree density ranged from 200 to 9600 trees ha^−1^ across plots, with a mean value of 3900, and 94.1% of plots had over 500 native trees ha^−1^ which is the target seedling planting number in assisted forest restoration in Indonesia. Native tree height, as estimated from DBH (Supplementary Fig. 8), has a mean value of 5.6 m overall which is the same as measured mean tree height (Table 2). Mean maximum tree height across all plots and species is 7.4 m (Table 2). However, for individual species, several trees reached a mean height of around or over 10 m and individual maximum heights over 15 m (Table 2, Supplementary Table 5), rivalling the mean height of 17.6 m for the remaining *A. crassicarpa* (Supplementary Table 5).

## Discussion

### Effectiveness of compacted peat dams for rewetting

Rewetting is an essential pre-requisite for the successful restoration of drained peatlands^19^. Drain or canal blocking using dams or other water control structures aims to minimize the impacts of drainage in order to reduce carbon emissions and peat subsidence whilst also providing conditions suitable for the restoration of the native peatland vegetation^20^. Given the relatively small dimensions of drainage features in northern peatlands (e.g. drains in UK upland blanket bogs are typically 50 cm deep × 50–70 cm wide^20^) drain blocks can be modest structures. In tropical peatlands, by contrast, the larger dimensions of canals (usually between 5 and 10 m wide and 3–5 m deep) combined with higher rainfall rates, require the use of larger, more substantial structures. In our study area, the use of compacted peat dams and their relatively high density at 500 m intervals resulted in small water level differences across most dams. Where canal blocking was most successful in rewetting the peatland, groundwater levels were raised from an estimated ~ − 0.6 m or below (based on limited pre-intervention observations) to ~ − 0.3 m on average. Dams without bypasses were found to perform best, as bypasses eroded rapidly which lowered water tables and increased canal peak flow velocities, which in turn resulted in limited canal vegetation growth. In fully blocked canals, the water surfaces were rapidly overgrown with aquatic vegetation. The extent of this vegetation cover suggests that in the long term, undisturbed canals may fully close up with vegetation and no longer drain the peatland. This process takes time and will eventually require growth of highly water tolerant trees. Planting of such trees along canal sides could speed up the process of rewetting. It is also worth noting that the creation of pools of standing water in canals and the revegetation of open water surfaces will likely enhance biodiversity support, particularly for invertebrates, as has been found in the restoration of temperate peatlands^21^.

The compacted peat dams applied in the rewetting trial to block canals were found to be easy to construct in a limited time (about 1 dam per day) and did not require maintenance in the absence of bypasses, being overgrown with vegetation quickly and still fully intact after 6–8 years. When built in large volumes, the construction cost per dam is estimated at ~ 300 US$, which is very economical compared to other rewetting methods using wood and other materials brought in from elsewhere.

While canal blocking is required for rewetting of drained peatland, it should be noted that rising water levels can be partly attributed to peat surface subsidence, bringing the surface closer to the water table rather than the other way around. Continued surface subsidence after the start of rewetting can therefore be seen as an inevitable and even necessary component of the rewetting process, bringing the peat-water system to a more sustainable balance.

### Subsidence and carbon emission reduction by rewetting

The major reduction in peat surface subsidence rate, from ~ 3–7 cm yr^−1^ (depending on reference period) in the plantation affected by road canal drainage to around 1.5 cm yr^−1^ in the part of the restoration area and formerly drained forest where water tables were raised the most, demonstrates that peat loss and hence carbon emission by peat decomposition can be substantially reduced within 5 years after canal blocking. As tropical domed peatlands tend to have rather uniform characteristics, consisting of fibric woody peat with a limited range of bulk density and carbon concentration^1,18^, it is possible to estimate carbon emission from subsidence rate applying default parameters. Following the approach and parameters demonstrated by Couwenberg and Hooijer^22^, each centimeter of reduced annual subsidence corresponds to a reduction in carbon emission by 4.3 Mg ha^−1^ yr^−1^. The range of 1.5 (observed) to 5.5 (deduced) cm yr^−1^ of subsidence rate reduction observed in the well-rewetted study area therefore indicates a carbon emission reduction of 6.4 to 23.6 Mg ha^−1^ yr^−1^.

It is worth noting that the reduction in subsidence rate in some locations lags the rising water table by months or even a few years. This may be explained by peat loss due to decomposition taking place from the upper zone of dried peat, not just from the peat surface. The top peat column will therefore gradually lose mass and structure to some depth, resulting in stepwise surface lowering occurring when bearing capacity is exceeded, e.g. by animal disturbance or after heavy rainfall saturating the surface peat and increasing its weight. In some cases we do indeed observe accelerating subsidence rate when water tables are rising after drought events. Moreover, the peat surface is sometimes observed to rise during prolonged periods of high water table, further demonstrating that part of the peat response to changes in water content is the temporary mechanical shrinking and swelling of the top peat. This is referred to as 'bog breathing', see also Sulaeman et al.^23^ and references therein.

This subsidence lag to water table change may also partly explain why some studies of CO_2_ carbon flux do not closely match subsidence measurements, especially where one or both sets of data are over a relatively short time period or have limited observation points. Another reason for discrepancies will be that subsidence measurements integrate all peat loss components (as CO_2_ and CH_4_ gases, and as Dissolved and Particulate Organic Carbon in water). However, where CO_2_ flux is the main carbon loss component and subsidence and CO_2_ flux are both measured carefully, over long time periods and a large number of locations, a good match is found for the two methods^18,24^.

In the study location, peatland rewetting has resulted in reduced subsidence rate (and probably emissions) not only from the retired plantation area but also from the adjoining PSF. Even in forest 1400–2100 m from canals, the mean subsidence rate was reduced from 1.6 cm yr^−1^ over the period 2016–2019 (during and after canal blocking, but excluding the 2019 drought), to 0.7 cm yr^−1^ over the period 2020–2023. This demonstrates that drainage impact can extend far from canals, as has been observed in other studies of peat subsidence^17^ and the carbon emission reduction benefit of rewetting may therefore also extend to a wider area beyond the intervention zone. However, it cannot be assumed in this least-drained forest that subsidence is caused by peat loss only (Supplementary Note 6) because a component of compaction and consolidation cannot be excluded here. In this situation, calculation of carbon emission from subsidence would likely result in an overestimation.

The major changes seen in the 7.5-year record of groundwater table depth and peat surface subsidence during the El Nino drought year of 2019 demonstrate that the condition of the restoring peatland hydrology and carbon balance are best understood based on a multi-year monitoring record. Had only data over 2019–2020 been available, the very low water table and high subsidence rate would have suggested extreme carbon loss. On the other hand, had only data over the last 3 years on record been available covering ‘average to wet’ years, water tables would have seemed high and stable while subsidence and carbon loss would have been underestimated.

The occurrence of subsidence and carbon emission even in unlogged PSF far from canals, that is closest to being ‘intact’, has been observed in some other areas of SE Asia e.g.^18,25,26^. This may be indicative of an overall disturbance of PSF systems in the SE Asian region. The causes may include drainage impact extending far from canals, but also a warming and drying climate in a region where climate change is expected to have particularly severe consequences^27,28^. A further potential cause may be a regional increase in atmospheric nutrient load, possibly linked to peatland and forest fires in recent years having caused major haze events that mobilized large amounts of hazardous chemicals that may have affected the peat surface microbiological and forest ecology balances^29^. Considering the long data record available, it is also evident that a severe drought could still cause major water table drops to near 1 m below the peat surface and associated carbon emission spikes even in otherwise ‘rewetted’ conditions, both in restoration areas and in PSF. This indicates that peat loss during droughts may be an inevitable feature of the remnant, disturbed tropical PSFs of SE Asia and suggests that the resilience to drainage of these systems may be decreasing^30^.

### Optimum rewetting conditions for forest regrowth

Field observations of the many PSF species returning to the rewetted former plantation in high numbers, combined with the absence of the *A. crassicarpa* plantation crop in 50% of plots 9 years after planting, confirm that unassisted forest restoration without tree planting is a viable approach that will eventually see a native PSF flora take over from the plantation crop cover (Supplementary Fig. 9). However, the finding that current height of native trees is 5.6 m on average, although with some species already reaching well over 10 m, also indicates that full restoration to something similar to the original natural ecosystem cannot be a rapid process and may take some decades.

This result should likely be considered as a ‘best case scenario’ as the observation plots are close to the forest, and so are relatively easily reached by seeds; this is also evident from the high proportion of animal-dispersed species encountered. Wijedasa et al.^31^, for the same area, report a decrease in regrowth species diversity and abundance further away from the forest, with highest numbers found in the first 500 m. Thus at greatest distance from the forest edge, ‘enrichment’ tree planting may still be required to facilitate timely forest regrowth and increase in tree species diversity^32–34^.

Moreover, it is worth noting that while native trees had 9 years to re-establish in the former plantation, after *A. crassicarpa* planting in 2012, only the last ~ 6 years of these were in fully rewetted conditions, with canal blocking proceeding from 2015 to 2017. Considering tree heights, it is probable that part of the native tree recruitment and growth started when water tables were lower. As restoration started just a few years after plantation development, it is even possible that some seeds or plant material for vegetative regeneration from the original forest were still in place, although this is likely of minor importance since most PSF tree seeds have relatively short dormancy^35^. The continued growth success after rewetting confirms that these native PSF tree species do well with high water tables, unlike *A. crassicarpa* and other dryland species. It should also be considered that the trial benefited from taking place in a plantation context. This meant that fire risk, which has hampered other restoration efforts in the region^5,34^, could be contained by prevention measures and limiting access to non-staff during dry periods. It also allowed the trial, while being very large scale, to be implemented fast and in a uniform manner. The scale itself is probably helpful to the outcome by reducing edge effects on vegetation and leakage of water.

If the aim of rewetting is defined as achieving a water table regime approaching that of natural PSF, then it is important to understand the natural regime in order to define appropriate targets. We found that water depths in the least drained forest, several kilometres away from drainage, were − 0.25 to − 0.2 m on average, with surface inundation occurring no more than a few weeks per year. Prolonged inundation was, therefore, avoided. This may have been critical in the early stages of revegetation by allowing the establishment of a range of native PSF tree species and would imply that preventing mean water tables above ~ − 0.2 m is important to successful tree growth.

The poor *A. crassicarpa* growth observed in the LiDAR data over areas of highest water table suggests that rewetting for PSF restoration requires a well-planned and gradual approach to raising water levels. Based on our findings it appears that PSF regrowth can be optimized by raising water levels enough to cause the original *A. crassicarpa* trees to topple when they grow tall, while avoiding the flooding that prevents establishment of most native species. Achieving this requires careful attention to the design and monitoring of canal blocking schemes.

## Conclusions

**Figure 6:**
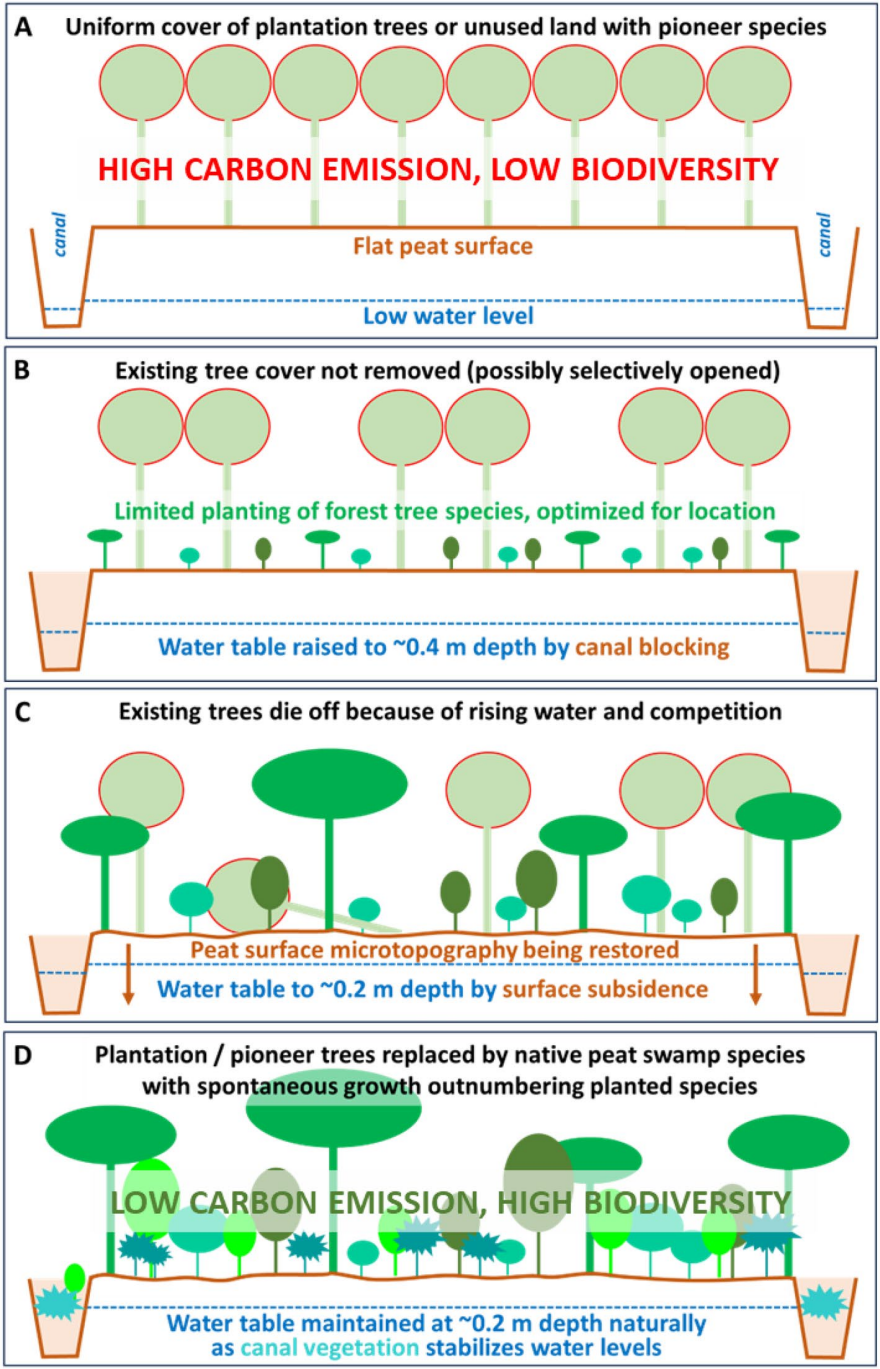
Visual summary of steps in restoration as identified in the study, highlighting the interactions between rewetting and peat swamp forest restoration, jointly resulting in nature-based carbon emission reduction and ecosystem enhancement.

We present the results from the initial seven years of a large-scale restoration trial in a deforested, drained tropical peatland. As far as the authors are aware, this is the first study of its kind from a region that has experienced widespread loss of native PSF and peatland drainage^2^ and associated carbon emissions^36^. We demonstrate that restoration through canal blocking has led to an enduring rise in the water table and initial recovery of the former vegetation cover through the re-establishment of native PSF tree species. The study results provide the basis for an outline strategy for stepwise nature-based PSF restoration and carbon emission reduction (Fig. 6). The only active interventions are [i] canal blocking for moderate rewetting that avoids inundation where possible, [ii] limited local removal of existing non-native tree cover, and [iii] possibly ‘enrichment’ planting of PSF species in areas far from remaining forest. Other beneficial changes to the system, i.e. unassisted native tree recruitment and forest regrowth, further peat surface adjustment by subsidence, and canal vegetation development to complete the blocking process, will occur spontaneously after the initial rewetting intervention. Taking a stepwise approach to restoration allows for monitoring of the system response to interventions, which may be different for different areas, and enables adjustments to optimize results. For instance, as we have seen in our trial, the initial rewetting plan may leave water tables in some areas too low and in others too high, which may be remedied by creating more dams or opening others after some years.

This approach is based on our findings in an area that was relatively close to remaining natural PSF, and where conditions allowed rewetting without causing inundation. Moreover, the peat in the area had not burnt. It is recognized that the outcome in some other areas may be less predictable, or may take a longer time to yield successful forest regrowth. Nevertheless, at our site, we believe that the results indicate the potential for the restoring peatland to reach a new optimum dynamic balance between peat decomposition rate, vegetation growth and water table depth. This will enable the highest possible success rate at the lowest possible cost, allowing peatland restoration at the large scale that is now required in SE Asia to achieve the goals of carbon emissions mitigation and forest biodiversity protection and enrichment.

According to the Spatial Database of Planted Trees^37^, planted tree plantations (Acacia and rubber, but mostly oil palm) on Sumatra, Borneo and Peninsular Malaysia cover 5.8 million ha on peat^38,39^, which will mostly have industrial scale canal water management similar to the TPJ restoration pilot area. The majority of these plantations are with 3.1 million ha (54%) located in Riau, Jambi and South Sumatra Provinces in Indonesia of which 1.2 million ha (37%) is on peat over 3 m in depth^40^. It follows that the potential for application of the rewetting method evaluated in our study could be several million hectare at least, should plantation owners aim to restore forest in parts or all of their peatlands. An important driver for retiring plantations and restoring peatland forest, apart from maintaining biodiversity and reducing carbon emissions, may be avoidance of crop loss due to flooding. The Round Table for Sustainable Palm Oil (RSPO) guidelines state that plantation companies are expected to conduct regular assessments of when surface subsidence will reduce drainability (relative to river and sea level) to the point that regular inundation becomes inevitable; when this point is 40 years away, such plantations are expected to be ‘phased out’^41^.

### Supplementary Information

Supplementary Information.

## Data Availability

The datasets analyzed during the study are available from the corresponding author on reasonable request.
