# Supplementary material for: Benefits of tropical peatland rewetting for subsidence reduction and forest regrowth: results from a large-scale restoration trial
Source: Sci Rep. 2024 May 10;14:10721. doi: 10.1038/s41598-024-60462-3 (PMC11087581; doi:10.1038/s41598-024-60462-3)
Supplement: Supplementary file 1 — Supplementary Information. [file 41598_2024_60462_MOESM1_ESM.pdf]

Supplementary Information for

Benefits of tropical peatland rewetting for subsidence reduction and forest regrowth: results from a large-scale restoration trial

A. Hooijer, R. Vernimmen, D. Mulyadi, V. Triantomo, Hamdani, M. Lampela, R. Agusti, S.E. Page, J. Doloksaribu, I. Setiawan, B. Suratmanto, S. Swarup

This document contains additional information used to inform our analyses. The following notes, figures, and tables are included:

• Supplementary Notes 1-6

• Supplementary Figures 1-9

• Supplementary Tables 1-5

Supplementary Note 1. Detailed Tables of water table measurements

Supplementary Table 1. Canal water levels for individual blocks along Canal #9, near the dipwell transect, from the main road canal where water levels remain lowest to the forest boundary. Values for representative wet and dry conditions (May 2023 and August 2023) are shown in Figure 2 in the main text.

Distance from road canal [m]	Block ID	Location	Feb-22	Jul-22	Aug-22	Sep-22	Oct-22	Nov-22	Feb-23	Apr-23	May-23	Jun-23	Jul-23	Aug-23	Sep-23
0	Canal		10.38	10.21	10.12	10.25	10.39	10.30	10.38	10.39	10.35	10.31	10.20	10.10	10.15
193	C9D1	W of block	10.38	10.21	10.12	10.25	10.39	10.30	10.38	10.39	10.35	10.31	10.20	10.10	10.15
193	C9D1	E of block	10.58	10.44	10.39	10.49	10.61	10.57	10.62	10.81	10.81	10.67	10.45	10.27	10.30
546	C9D2	W of block	10.58	10.44	10.39	10.49	10.61	10.57	10.62	10.81	10.81	10.67	10.45	10.27	10.30
546	C9D2	E of block	10.58	10.43	10.40	10.49	10.60	10.57	10.61	10.89	10.90	10.76	10.57	10.37	10.40
1090	C9D3	W of block	10.58	10.43	10.40	10.49	10.60	10.57	10.61	10.89	10.90	10.76	10.57	10.37	10.40
1090	C9D3	E of block	10.74	10.59	10.49	10.63	10.72	10.76	10.75	10.96	10.93	10.80	10.66	10.47	10.47
1582	C9D4	W of block	10.74	10.59	10.49	10.63	10.72	10.76	10.75	10.96	10.93	10.80	10.66	10.47	10.47
1582	C9D4	E of block	10.76	10.65	10.52	10.66	10.75	10.80	10.80	10.98	10.93	10.81	10.67	10.53	10.49
2035	C9D5	W of block	10.76	10.65	10.52	10.66	10.75	10.80	10.80	10.98	10.93	10.81	10.67	10.53	10.49
2035	C9D5	E of block	10.77	10.67	10.53	10.66	10.76	10.82	10.81	10.99	10.94	10.83	10.69	10.58	10.49
2312	C9PD	W of block	10.77	10.67	10.53	10.66	10.76	10.82	10.81	10.99	10.94	10.83	10.69	10.58	10.49
2312	C9PD	E of block	10.77	10.68	10.54	10.67	10.73	10.81	10.81	10.99	10.94	10.83	10.69	10.57	10.48

Supplementary Table 2. Peat groundwater table depths (GWD) and subsidence rates for individual measurement points along the transect through the restoration area, from the main road canal where drainage remains most severe to forest that is ~2 km from any drainage. Values summarized by zones are presented in Table 1 in the main text.

**Distance from main road canal [m]:**		50	300	800	1050	1300	1550	1800	1950	2100	2125	2175	2200	2225	2500	2650	2900	3150	3650	3900	4150
**Distance from former plantation into forest [m]:**												25	50	75	350	500	750	1000	1500	1750	2000
**Dipwell code:**		B18-N1	B18-N2	B18-N4	B18-N5	B18-N6	B18-N7	B18-N8	B18-N9	B18-N10	B18-N11	B18-N12	B18-N13	B18-N14	B18-N19	B18-N20	B18-N21	B18-N22	B18-N24	B18-N25	B18-N26
**Total number of measurements (monthly, minus gaps):**		69	87	87	69	87	87	63	87	86	78	87	78	69	87	69	87	69	69	87	68
**Zone along transect:**		**Least rewetted restoration (road canal)**		**Intermediate restoration**			**Well rewetted restoration**					**Somewhat drained forest**					**Intermedia te forest**		**Least drained forest**		
**Water table depth below peat surface (m)**																					
Mean 2016 - mid 2023 (7.5 yr)		-0.67	-0.61	-0.49	-0.47	-0.61	-0.48	-0.38	-0.37	-0.26	-0.28	-0.33	-0.25	-0.33	-0.37	-0.47	-0.29	-0.20	-0.21	-0.23	-0.29
Mean2016 - mid 2019 (3.5 yr)	*Rewetting started*	-0.56	-0.61	-0.46	-0.42	-0.59	-0.44	-0.32	-0.33	-0.27	-0.33	-0.35	-0.27	-0.34	-0.34	-0.49	-0.27	-0.20	-0.24	-0.24	-0.30
Mean mid 2019 - mid 2020 (1 yr)	*El Nino drought*	-0.83	-0.91	-0.78	-0.78	-0.93	-0.77	-0.86	-0.68	-0.40	-0.18	-0.44	-0.16	-0.51	-0.52	-0.61	-0.43	-0.37	-0.38	-0.35	-0.35
Mean mid 2020 - mid 2023 (3 yr)	*Rewetting improved*	-0.69	-0.50	-0.42	-0.38	-0.51	-0.42	-0.14	-0.32	-0.19	-0.23	-0.26	-0.23	-0.24	-0.34	-0.41	-0.26	-0.13	-0.12	-0.18	-0.27
Minimum monthly value (7.5 yr)		-1.18	-1.35	-1.33	-1.69	-1.80	-1.41	-1.72	-1.56	-1.00	-0.81	-0.90	-0.58	-0.97	-1.01	-1.01	-0.94	-0.90	-0.91	-0.81	-0.80
Maximum monthly value (7.5 yr)		-0.27	-0.19	-0.02	-0.05	-0.22	-0.11	-0.05	0.00	0.10	-0.01	-0.06	0.59	-0.04	-0.12	-0.18	0.01	0.06	0.11	0.03	0.06
Standard deviation (7.5 yr)		0.18	0.22	0.22	0.29	0.26	0.24	0.33	0.26	0.17	0.13	0.15	0.15	0.20	0.17	0.17	0.18	0.20	0.21	0.17	0.17
Median 2016 - mid 2023 (7.5 yr)		-0.68	-0.60	-0.47	-0.43	-0.59	-0.46	-0.29	-0.34	-0.23	-0.28	-0.32	-0.26	-0.29	-0.34	-0.46	-0.28	-0.17	-0.19	-0.23	-0.28
10% 2016 - mid 2023 (7.5 yr)		-0.85	-0.87	-0.68	-0.63	-0.81	-0.70	-0.62	-0.53	-0.44	-0.45	-0.48	-0.41	-0.58	-0.53	-0.68	-0.44	-0.39	-0.41	-0.41	-0.49
90% 2016 - mid 2023 (7.5 yr)		-0.46	-0.37	-0.29	-0.21	-0.35	-0.25	-0.13	-0.14	-0.08	-0.14	-0.17	-0.12	-0.11	-0.19	-0.27	-0.10	-0.01	0.02	-0.04	-0.11
**Peat surface subsidence (cm yr^{-1})**																					
Mean 2016 - mid 2023 (7.5 yr)		7.3	6.8	4.7	4.4	5.0	3.5	2.8	3.7	2.9	2.9	3.2	3.4	3.0	1.8	2.7	2.5	2.2	3.1	2.4	1.3
Mean 2016 - mid 2019 (3.5 yr)	*Rewetting started*	8.0	6.2	4.1	3.7	5.1	2.7	3.7	3.2	2.9	3.1	3.9	4.7	4.3	1.8	1.8	2.1	2.5	2.0	1.0	1.9
Mean mid 2019 - mid 2020 (1 yr)	*El Nino drought*	16.6	19.7	15.3	9.6	7.2	13.8	1.8	12.5	8.3	6.1	6.3	5.2	5.0	4.7	8.0	9.3	6.0	16.0	10.0	2.0
Mean mid 2020 - mid 2023 (3 yr)	*Rewetting improved*	3.3	3.3	1.9	3.4	4.2	1.0	2.1	1.3	1.1	1.7	1.2	1.1	0.8	0.8	2.0	0.7	0.6	0.2	1.7	0.3

Supplementary Note 2. Field surveys to link canal water depth (CWD) to groundwater table depth (GWD)

A field survey was conducted to determine the difference between water levels as measured in main canals (from staff gauges and from LiDAR data) with those measured in the peat (from dipwells). This was done using levelling instruments. Water level elevation in 11 dipwells in the restoration area was linked to that in the nearest canal, either to the south or to the North of the transect. The greatest distance between dipwell and canal was 225 m, the mean distance was 105 m. The mean difference between canal water level and ground water level was 5 cm with a standard deviation of 7 cm. This value of 5 cm is used to calculate ground water level from canal water level in our study.

The survey was conducted by July 2023, in a period with relatively low water levels. It may be that the difference between canal and ground water levels in wet periods is somewhat greater. However, from observations we do not expect the overall difference to exceed 10 cm, in conditions where canals are closely spaced (at 500 m, with field drains at ~100-200 m) and peat hydraulic conductivity is high, allowing rainfall to be discharged from the peat quickly.

Supplementary Note 3. Peat hydraulic conductivity measurements

Hydraulic conductivity was measured using the Hooghoudt auger hole method[1]. While this method originally was developed for a borehole with a diameter of approximately 10 cm's, we carried out the measurements in the square soil pits constructed for peat sampling (Supplementary Note 6).

Since the measurement assumes no-flow conditions at the start of the measurement, the water table in the soil pit should first reach equilibrium with the groundwater level. After the soil pit was dug, the water inside the pit was quickly pumped out of the pit (as was also required for the peat sampling). After pumping, the groundwater begins to seep into the soil pit again and the rate at which it rises is measured. The hydraulic conductivity is then derived by a relation between the rise of the water table, the groundwater conditions and the geometry of the soil pit.

During the measurement a lowering of the groundwater table near the soil pit will develop, because of the lowering of the water table in the soil pit. This lowering of the groundwater table will develop gradually. The theory assumes a flat groundwater table around the soil pit. Therefore, the measurements cannot be continued to long after the lowering of the water table in the hole. During the measurement the water in the soil pit must not rise by more than a quarter of the bailed out length. In other words, if you lower the water table with 80 cm's the measurements should be stopped once the water level has risen with 20 cm's.

The hydraulic conductivity is calculated as follows using the empirical equation of Ernst[2]:

$$K = C * (\mathrm{dy} / \mathrm{dt}) \qquad \text{[Eq. 1]}$$

Where:

K = hydraulic conductivity (m d^{-1})

C = geometry factor (-)

dy = vertical distance between two subsequent measurements (cm)

dt = time (s)

The geometry factor can be derived by the following Ernst equation which is valid when $S > 6r$, or in other words when using a soil pit of 1 x 1 m peat depth has to be at least 6 * 0.5 or 3 m

$$C = [4000 * r / y'] / [(20 + H/r)\ (2 - y'/H)] \qquad \text{[Eq. 2]}$$

Where:

H = wet length of the soil pit (cm)

y' = vertical distance between the groundwater level and average level between two subsequent measurements (cm)

r = radius of the soil pit (cm)

S = distance between the lower end of the soil pit and the next soil layer (cm)

The resulting hydraulic conductivity values are presented in Supplementary Table 3.

Supplementary Table 3. Hydraulic conductivity as determined by the Hooghoudt auger hole method applied in 5 soil pits in the restoration area. Water table depth (WTD) and the number of replicate measurements (n) is also provided.

Date	Peat thickness	Pit depth	Pit width	Pit length	WTD	n	Hydraulic conductivity
	[m]	[m]	[m]	[m]	[m]		[m d^{-1}]
13-Aug-22	10.2	2.50	0.99	0.92	0.47	3	113.6
15-Aug-22	10.2	2.59	0.78	0.79	0.56	3	163.4
10-Sep-22	10.2	2.50	1.38	1.21	0.40	3	118.7
24-Sep-22	10.1	2.58	1.10	1.22	0.64	3	112.7
25-Sep-22	10.1	2.79	1.13	1.03	0.55	3	86.3
**Mean**	**10.2**	**2.59**	**1.08**	**1.03**	**0.52**		**119.0**

Supplementary Note 4. Peat surface subsidence in relation to water table depth

We find a clear relation between groundwater depth and subsidence rate (Supplementary Fig. 1), similar to those found in earlier studies. However, relations are somewhat different when taking data from the entire 7.5-year record or from shorter parts of it. Comparison of the first 3.5 and last 3 years on record (excluding the 2019-2020 drought event) suggests a shift in the relation, towards a ~25% lower subsidence rate at a given water depth.

It was demonstrated by Hooijer et al.[3] that it may take up to 5 years after initial plantation drainage before subsidence rates stabilize, from then on being caused by peat decomposition alone[4] with negligible contributions by compaction and consolidation[5]. As the 2016-2019 record starts some 5 years after plantation establishment in 2011, a minor contribution of compaction may explain some of the ~20% difference with the 2020-2023 record, apart from the inaccuracies that are inherent in field measurements of this nature. On the other hand, having a long record of GWD and subsidence will reduce the relative effect of measurement errors, as is indicated by the relatively high R^2 value of 0.67 for the 7.5-year record (Supplementary Fig. 1). The 7.5-year record also includes a greater variation of climate conditions including the 2019 El Nino drought, making it most representative of long-term conditions. In further calculations of the benefits of raising water levels in this area, we will therefore apply this relation found over the 7.5-year record to represent long-term conditions including extreme drought events (Supplementary Fig. 1):

$$Subsidence = -9.04*GWD + 0.29 \qquad [cm\ yr^{-1}] \qquad \text{[Eq. 3]}$$

Where GWD is groundwater depth *[-m; negative]*

However, the following relation found over the 2020-2023 data record will be applied to calculate a minimum estimate of subsidence at a given water depth, as it represents the most stable conditions 10 years after drainage without compaction impacts (as is also evident from bulk density profiles in the study area, see Supplementary Note 6):

$$Subsidence = -6.21*GWD + 0.08 \qquad [cm\ yr^{-1}] \qquad \text{[Eq. 4]}$$

This latter relation is very close to the relation reported by Hooijer et al.[3] for forest that is affected by plantation drainage, which was also measured over several years without a drought event; we consider this the most conservative relation.

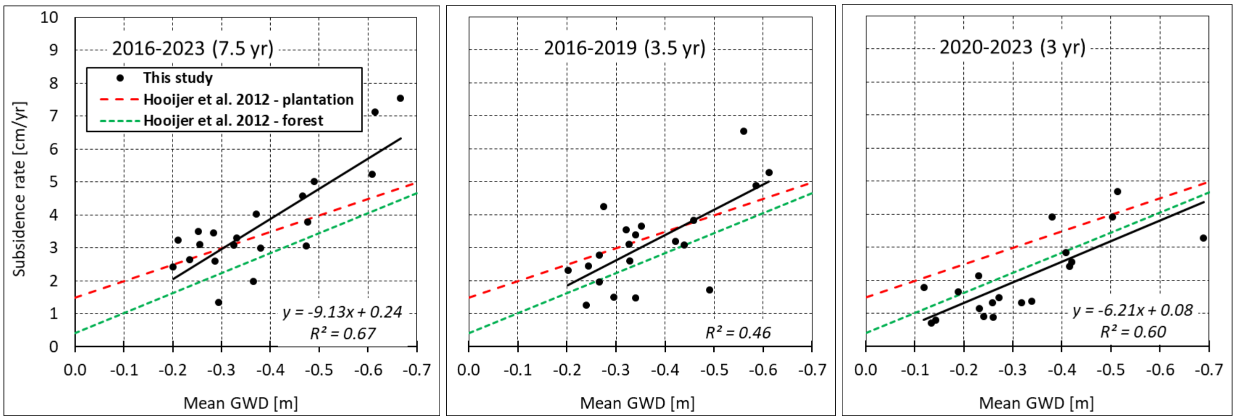

Supplementary Fig. 1. Peat surface subsidence as a function of groundwater table depth, for the entire 7.5-year measurement record and for the first 3.5 and last 3 years. A similar relation from an earlier study is shown for reference[3].

Supplementary Note 5. Relation between *A. crassicarpa* canopy growth and GWD

The relation between vegetation growth and GWD, as determined for the LiDAR (Fig. 1, Fig. 5), confirms that growth is lowest where water tables are highest i.e. where the peat is most often saturated to the surface or inundated (Supplementary Fig. 2). The relation between the two is strongest after 1.5 years ($R^2 = 0.56$) but weaker after 3.5 years ($R^2 = 0.40$). This may be due partly to the GWD values being an indirect estimate based on only 4 canal water depth measurements in time. However, it may also indicate that A. *crassicarpa* after initially slow development, may eventually grow fast in all but the wettest areas.

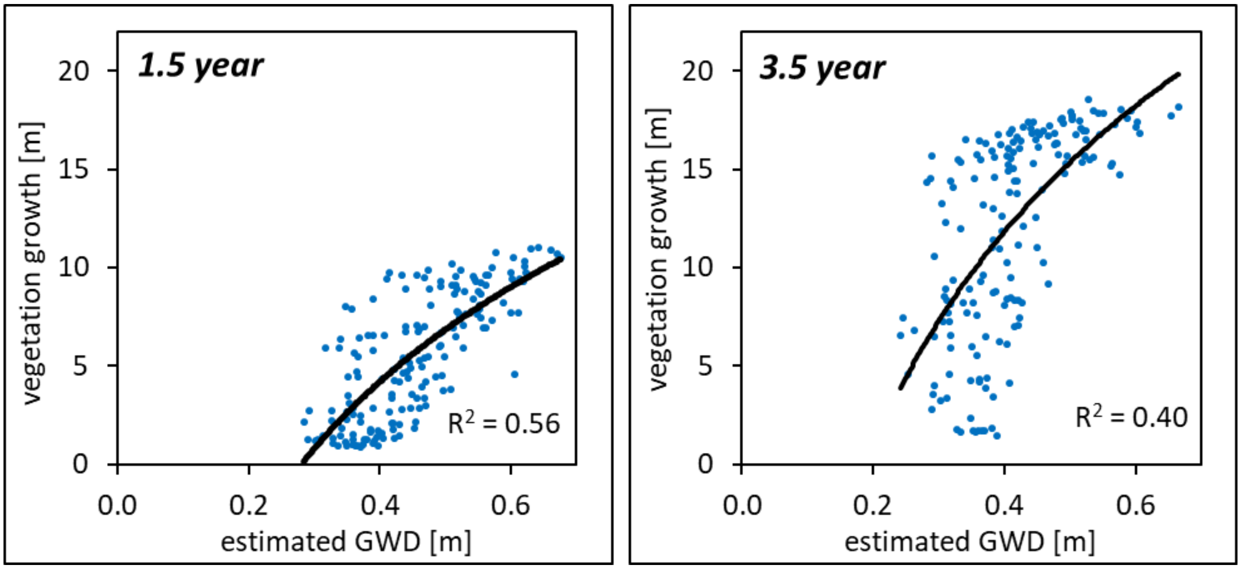

Supplementary Fig. 2. Median vegetation height increase of spontaneous regrowth of mostly *A. crassicarpa*, after ~1.5 year (August 2018) and ~3.5 year (July 2020) since first measurement by LiDAR (January 2017) after plantation cover removal (March – May 2016) as affected by spatial variation in estimated groundwater depth (GWD; mean value over 4 2017-2020 observations) over the same period for the area shown in Fig. 5. Data points (n = 171) correspond to 100 m grid cells, excluding grid cells that overlap canals or the adjoining unharvested area to minimize edge effects in the data. Note that GWD is presented here as a positive value to allow the logarithmic relationship to be derived.

Supplementary Note 6. Bulk density profiles across the restoration area

Bulk density was measured in peat pits (Supplementary Fig. 3), a method that yields the most accurate results by ensuring minimum sample disturbance[3], compared to measurements of auger samples that tend to be more disturbed.

Peat pits of ~1*~1 m width were dug up to almost 3 m depth in the dry season when water tables were low. Incoming water was removed by pumping. Peat samples were collected at 10 cm depth intervals in the top layer to 0.4 m depth, at 0.2 m intervals below it; 3 replicate samples were taken at each depth to a total depth of 2.4 m. In the wet season, pits were less deep and sampling was possible to depths of only 1.2 m. Steel rings of 7.2 cm diameter and 4.5 cm length were used for sampling, sample disturbance was minimized by having sharpened ring edges. Disturbance of samples was avoided during handling, transport and storage of samples. Samples of 183 cm^3 in volume were dried at 105 degrees in a laboratory and weighed at 12, 24 and 36 hours to ensure no moisture was left. Remaining dry weight after 36 hours was divided by the sample volume to determined bulk density in units of g cm^{-3}. This approach was described in more detail, by Hooijer et al.[3].

The resulting data from 17 pits and 534 samples are presented in Supplementary Fig. 4, as profiles against depth. As sample disturbance could not be avoided completely and did cause a few outliers, median values were calculated over the 3 replicates per depth, and then over all pits in each landcover type: restoration area (former plantation; 9 pits), somewhat drained forest less than 700 m from the perimeter canal between the restoration area and forest (4 pits), and less drained forest at 700–1000 m from the perimeter canal (4 pits); sampling further than 1000 m from the canal was not possible in this case for logistical reasons).

Bulk density profiles in the restoration area (i.e. rewetted former plantation) and somewhat drained forest that is less than 700 m from the former plantation are very similar, with values of 0.124–0.132 g cm^{-3} at 0.1 m depth below the peat surface declining to 0.062 g cm^{-3} at 2–2.4 m depth. In forest further away from drainage, bulk density values are somewhat lower at all depths, starting at 0.109 g cm^{-3} at 0.1 m depth (Supplementary Table 4, Supplementary Fig. 4). This pattern is consistent with the peat in the restoration area and in somewhat drained forest having been subjected to compaction and compression processes during the initial plantation development phase that was accompanied by severe drainage to 'dewater' the peat and ensure surface stability for access and planting[5]. As these processes are temporary and the peat density stabilizes at some point, further peat surface subsidence in these zones can therefore be attributed to peat loss due to decomposition[4,5]. In the less drained forested peat however, past compaction and compression have been less severe and may still be ongoing in dry periods, possibly causing some of the subsidence measured there.

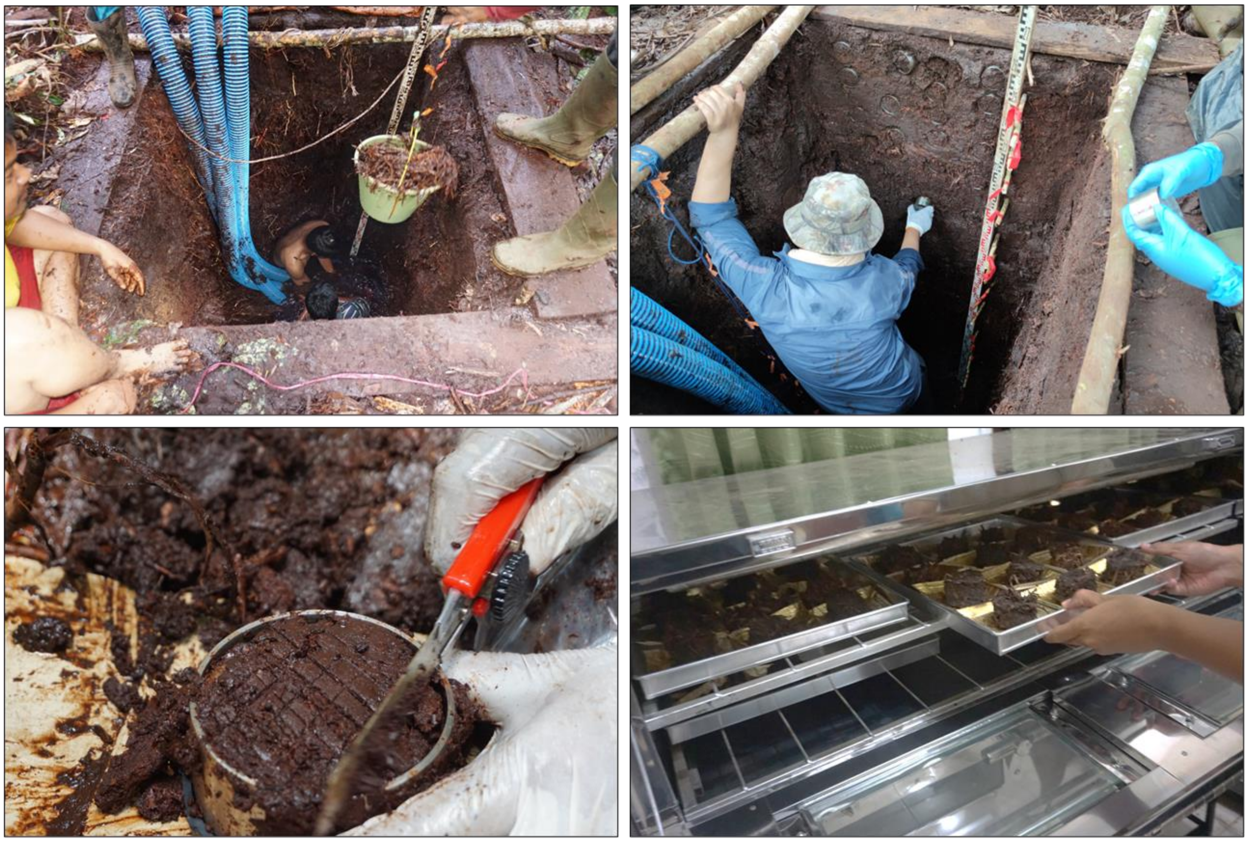

Supplementary Fig. 3. Photos of peat pit sampling. Clockwise from top left: digging the pit while pumping water; sampling in completed pit; sample preparation in the steel ring; trays with peat samples prepared for drying.

Supplementary Table 4. Summary results of bulk density sampling in peat pits.

	**Bulk density (median and SD values over sampling pits)**					
	Restoration area		Forest < 700m		Forest > 700m	
Number of locations:	*9*		*4*		*4*	
**Sample depth** *[m]*	Median *[g cm^{-3}]*	SD *[g cm^{-3}]*	Median *[g cm^{-3}]*	SD *[g cm^{-3}]*	Median *[g cm^{-3}]*	SD *[g cm^{-3}]*
0.1	0.124	0.037	0.132	0.012	0.109	0.023
0.2	0.119	0.031	0.109	0.025	0.092	0.016
0.3	0.098	0.024	0.090	0.019	0.078	0.012
0.4	0.074	0.016	0.088	0.010	0.062	0.011
0.6–0.8	0.073	0.012	0.070	0.012	0.067	0.009
1–1.2–1.4	0.071	0.013	0.069	0.015	0.066	0.007
1.6–1.8	0.067	0.012	0.066	0.007	—	—
2–2.2–2.4	0.062	0.008	0.062	0.004	—	—

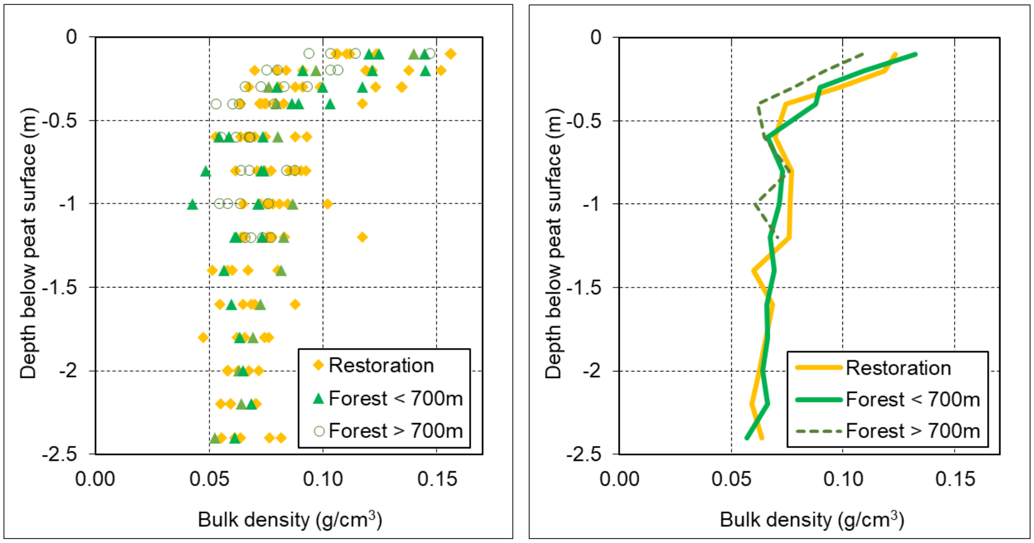

Supplementary Fig. 4. Results of the peat pit bulk density measurements in the study area. Left: each point representing the median value of 3 replicate samples at each depth in individual pits. Right: median values over all peat pit locations in one land cover zone.

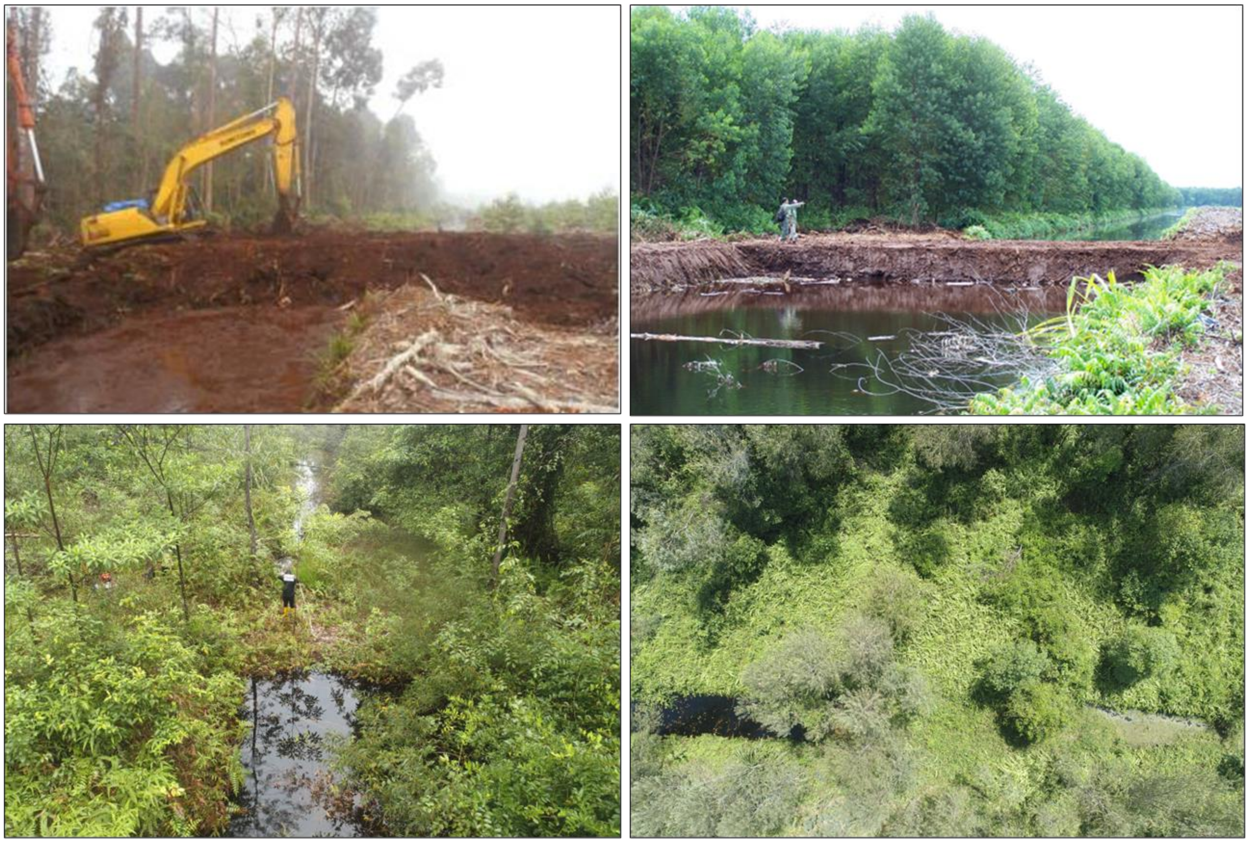

Supplementary Fig 5. Compacted peat dams. Clockwise from top left: dam construction in the restoration area (2015-2016); recently completed dam pushing up the canal water table; dam after ~6 years. overgrown by vegetation; aerial view of vegetated dam (drone image).

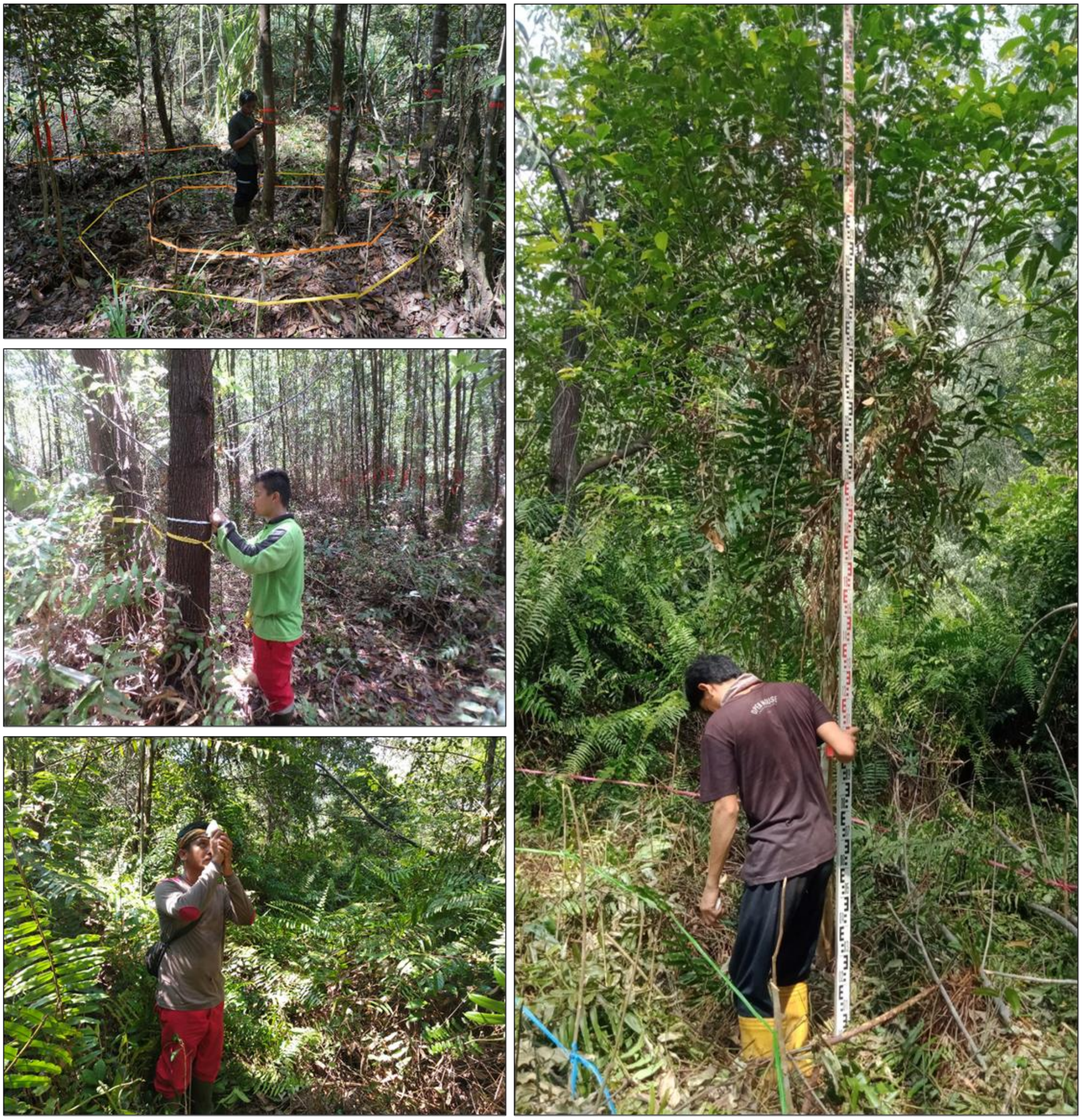

Supplementary Fig. 6. Vegetation surveys. Top to bottom, left to right: delineation of circle plots; measuring diameter of tree using a diameter tape; measuring height of tree using a clinometer; measuring height of tree using a levelling rod.

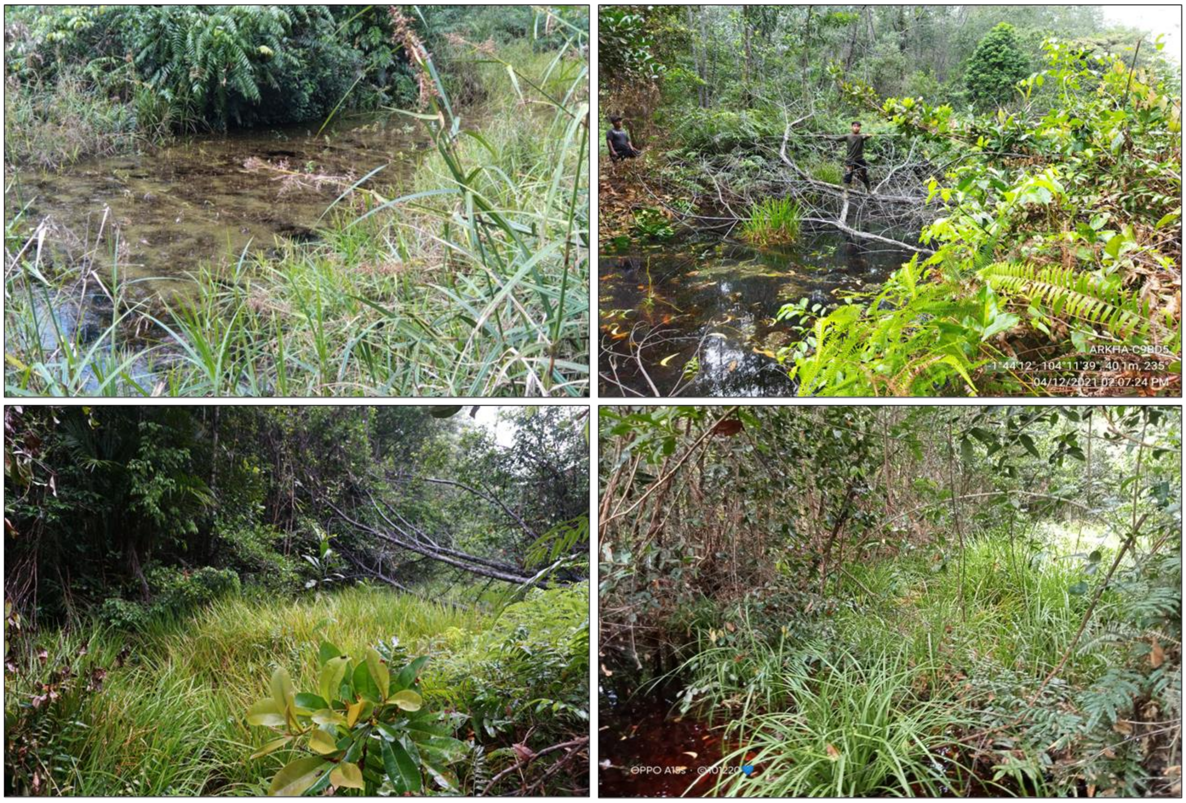

Supplementary Fig. 7. Stages of canal vegetation succession after ~6 years. Clockwise from top left: floating vegetation; clumps of aquatic sedges and fallen trees; canal covered with sedges and grasses; woody shrubs invading the canal.

Supplementary Table 5. Key parameters for the native tree regrowth species and *A. crassicarpa*, occurring in 34 rewetted plots (~9 years after *A. crassicarpa* planting, ~5 years after rewetting).

Species name		Wind dispersed	Water dispersed	Bird dispersed	Mammal dispersed	Occurring in # plots		Total number of stems	Total number of trees	Mean tree density	Mean stem diameter	Max. stem diameter	Height measurements	Mean tree height	Max. tree height
**Latin**	**Local (Indonesia)**					*[%]*	*[#]*	*[#]*	*[#]*	*[# ha^{-1}]*	*[cm]*		*[#]*	*[m]*	
Acacia crassicarpa	Akasia	1	0	0	0	50	17	36	31	182	25.8	42.8	20	17.6	30.9
Actinodaphne sesquipedalis	n.a.	0	0	1	1	6	2	5	3	18	2.0	2.9	2	3.9	5.0
Alseodaphne insignis	n.a.	0	0	1	0	6	2	2	2	12	2.8	3.2	2	4.2	4.3
Alstonia angustifolia	Tembesu angin / Pulai pipit	1	0	0	0	38	13	73	50	294	7.1	16.0	21	9.1	15.0
Alstonia spatulata	Pulai putih, pulai gabus	1	0	0	0	3	1	1	1	6	2.4	2.4	1	3.5	3.5
Antidesma coriaceum	Mata udang	0	0	1	1	9	3	6	3	18	2.2	3.2	2	8.3	9.0
Ardisia pterocaulis	Pari	0	0	1	1	9	3	14	5	29	1.8	2.8	6	4.1	5.5
Artocarpus griffithii	Tampang / Nangka-nangka	0	0	1	1	12	4	7	5	29	6.8	10.2	4	9.6	13.0
Artocarpus sp	n.a.	0	0	1	1	3	1	1	1	6	7.8	7.8	1	2.0	2.0
Brackenridgea palustris	Keladang	0	0	1	1	9	3	5	4	24	2.6	4.4	3	4.8	7.0
Campnosperma auriculatum	Terentang / Ketapang	0	0	1	1	3	1	4	1	6	6.6	8.0	1	15.0	15.0
Canarium pilosum	Kanari hutan	0	0	0	1	3	1	3	1	6	3.8	4.2	1	5.5	5.5
Castanopsis foxworthyi	Berangan	0	0	0	1	3	1	11	9	53	7.2	11.3	2	11.3	11.6
Cratoxylum arborescens	Geronggang	1	0	1	0	6	2	6	4	24	3.7	8.3	3	6.3	10.0
Cryptocarya impressa	Medang api-api	0	0	1	1	3	1	1	1	6	5.6	5.6	1	9.0	9.0
Dacryodes rostrata	Kedondong hutan	0	0	0	1	9	3	4	3	18	3.1	3.6	3	5.7	7.0
Dillenia pulchella	Simpur paya	0	0	0	1	3	1	1	1	6	3.5	3.5	1	5.0	5.0
Diospyros areolata	Arang-arang	0	0	0	1	3	1	1	1	6	1.4	1.4	1	3.0	3.0
Diospyros baloen-idjoek	Arang-arang daun kecil	0	0	1	1	9	3	4	4	24	1.4	2.5	2	2.8	2.8
Diospyros pilosanthera	Arang-arang	0	0	1	1	9	3	3	3	18	1.5	1.6	3	2.9	3.5
Diospyros siamang	Arang-arang	0	0	0	1	6	2	2	2	12	6.1	8.2	2	9.0	13.0
Disepalum rawagambut	Pisang-pisang	0	0	1	1	3	1	5	1	6	4.3	6.2	2	8.5	9.0
Dyera polyphylla	Jelutung	1	0	0	0	6	2	2	2	12	2.4	3.6	2	3.3	5.0
Elaeocarpus acmocarpus	Getuk-getuk	0	0	1	1	21	7	14	11	65	5.3	13.0	10	6.1	10.0
Elaeocarpus griffithii	Garam-garam	0	0	1	1	29	10	18	12	71	4.6	8.7	12	5.3	9.5
Elaeocarpus mastersii	Karamunting	0	0	1	1	44	15	90	54	318	7.9	18.2	26	10.8	19.8
Ficus spathulifolia	Aro / Ara	0	0	1	1	3	1	1	1	6	8.0	8.0	n.m	n.m.	n.m.
Glochidion lutescens	Samak	0	0	1	1	3	1	1	1	6	2.6	2.6	1	5.3	5.3
Gluta aptera	Rengas	1	0	0	1	47	16	53	42	247	3.2	8.2	19	4.9	9.0
Gymnacranthera farquhariana	n.a.	0	0	1	1	6	2	3	2	12	1.6	2.3	2	2.8	3.5
Ilex cymosa	Kapesan	0	0	1	1	35	12	27	19	112	2.7	5.8	14	4.0	6.5
Ilex hypoglauca	Mengkulat	0	0	1	1	50	17	37	27	159	2.6	5.1	16	3.7	6.0
Knema laurina	Medang darah	0	0	1	1	6	2	3	3	18	7.2	9.7	2	6.8	10.0
Koompassia malaccensis	Kompas, Kempas	1	1	0	0	3	1	3	3	18	4.8	7.6	3	7.4	10.5
Maasia hypoleuca	Pisang-pisang daun kecil	0	0	1	1	3	1	1	1	6	1.3	1.3	1	3.0	3.0
Macaranga caladiifolia	Mahang semut	0	0	1	1	12	4	10	8	47	6.8	24.5	4	5.1	11.3
Madhuca motleyana	Balam	0	0	1	1	26	9	33	21	124	2.1	4.7	14	4.0	7.0
Mangifera parvifolia	Mangga hutan	0	0	0	1	3	1	1	1	6	2.7	2.7	1	5.5	5.5
Mezzettia havilandii	Pisang-pisang	0	0	0	1	3	1	1	1	6	4.2	4.2	1	5.5	5.5
Ormosia bancana	Duku-duku	0	0	1	1	24	8	43	28	165	3.5	8.6	17	5.3	10.0
Parartocarpus venenosa	Kulus	0	0	1	1	3	1	1	1	6	3.4	3.4	1	4.0	4.0
Pellacalyx axillaris	Bakau darat	0	0	1	0	6	2	2	2	12	4.5	7.5	2	3.3	3.9
Planchonella maingayi	Nyatoh babi	0	0	0	1	47	16	68	54	318	3.7	13.6	24	6.2	12.0
Ploiarium elegans	Jonger, Cerucuk	1	0	0	0	12	4	7	7	41	3.5	5.0	4	6.6	8.0
Psydrax dicoccos	n.a.	0	0	1	1	3	1	1	1	6	1.7	1.7	1	4.0	4.0
Ridsdalea grandis	Timun-timun	0	0	0	1	65	22	69	53	312	2.8	6.0	27	4.7	7.0
Shorea hemsleyana	Meranti kasar	1	0	0	1	3	1	1	1	6	7.0	7.0	n.m	n.m.	n.m.
Shorea teysmanniana	Meranti daun kecil	1	0	0	1	3	1	1	1	6	7.6	7.6	1	7.5	7.5
Simaba borneensis	Pait-pait	0	0	1	1	3	1	4	2	12	2.3	3.8	1	4.8	4.8
Syzygium bankense	Nasi-nasi	0	0	1	1	3	1	1	1	6	1.4	1.4	1	3.5	3.5
Syzygium grande	Jambu-jambu / Jambu	0	0	1	1	6	2	3	3	18	3.0	4.7	2	6.0	7.0
Syzygium incarnatum	Jambu-jambu	0	0	1	1	53	18	93	65	382	3.0	6.8	27	5.2	10.0
Syzygium napiforme	Kayu batu / Jambu-jambu	0	0	1	1	6	2	11	6	35	3.0	4.8	3	5.9	8.0
Syzygium urceolatum	Jambu-jambu / Jambu	0	0	1	1	18	6	13	8	47	2.3	4.9	7	4.1	6.0
Ternstroemia magnifica	Batu-batu	1	0	1	0	3	1	1	1	6	1.2	1.2	1	2.0	2.0
Tetractomia tetrandra	Medang rawang	0	0	1	1	3	1	2	1	6	1.9	2.2	1	6.0	6.0
Timonius flavescens	Berumbung	0	0	1	0	59	20	108	46	271	2.5	5.4	28	5.3	8.5
Tristaniopsis merguensis	Pelawan putih	0	0	1	1	29	10	33	17	100	2.7	4.5	12	6.0	9.0
**TOTAL (minus *A. crassicarpa*):**	**57**	**10**	**1**	**39**	**47**	**-**	**-**	**919**	**643**	**3600**	**3.8**	**6.1**	**352**	**5.6**	**7.4**

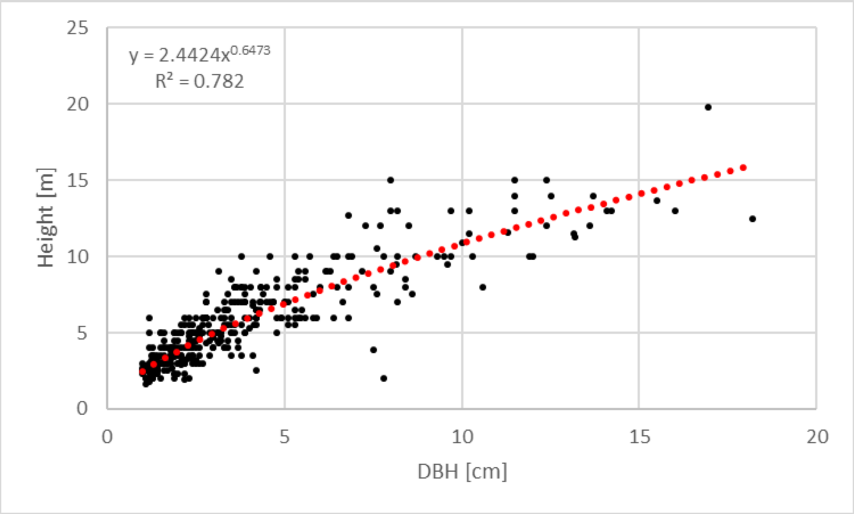

Supplementary Fig. 8. Diameter at breast height (DBH) vs height for native tree species. For 352 (38.3%) of the 919 native tree stems surveyed both DBH and tree height was measured (Supplementary Table 5).

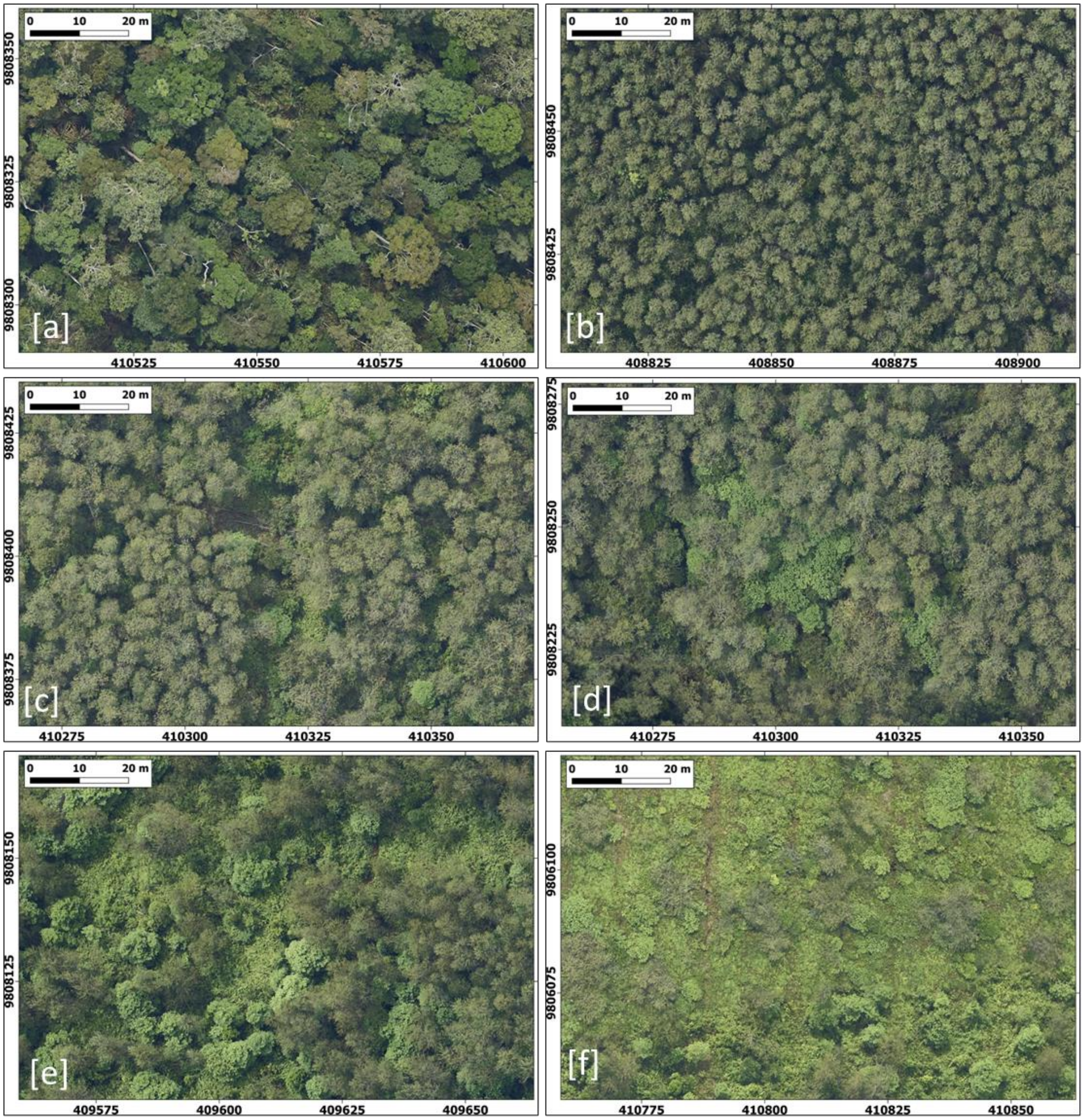

Supplementary Fig. 9. Orthophotos acquired in July 2020 over the restoration area and nearby peat swamp forest. [a] peat swamp forest, [b] *A. crassicarpa* plantation, [c] fallen *A. crassicarpa* trees and some regrowth, [d] much regrowth in between *A. crassicarpa*, [e] some *A. crassicarpa* and much regrowth, and [f] few *A. crassicarpa* trees and little regrowth. [a] - [e] are located along the monitoring transect (Fig. 1), [f] is located in the LiDAR study area (Fig.1).

References

1. van Beers, W. F. J. The auger hole method. A field measurement of the hydraulic conductivity of soil below the water table. ILRI publication 1, International Institute for Land Reclamation and Improvement (ILRI), Wageningen, 32 pp. (1958).
2. Ernst, L. F. A new formula for the calculation of the permeability factor with the auger hole method. TNO Groningen 1950. Translated from the Dutch by H. Bouwer, Cornell Univ. Ithaca, N.Y., 1955. (1950).
3. Hooijer, A. et al. Subsidence and carbon loss in drained tropical peatlands. Biogeosciences **9**, 1053–1071 (2012).
4. Couwenberg, J. & Hooijer, A. Towards robust subsidence-based soil carbon emission factors for peat soils in south-east Asia, with special reference to oil palm plantations. Mires and Peat **12: Art. 1**, (2013).
5. den Haan, E. J., Hooijer, A. & Erkens, G. Consolidation settlements of tropical peat domes by plantation development. Deltares Report 1202415, 41 pages. (2012).
